# Estimating spatial variation in the effects of climate change on the net primary production of Japanese cedar plantations based on modeled carbon dynamics

**DOI:** 10.1371/journal.pone.0247165

**Published:** 2021-02-17

**Authors:** Jumpei Toriyama, Shoji Hashimoto, Yoko Osone, Naoyuki Yamashita, Tatsuya Tsurita, Takanori Shimizu, Taku M. Saitoh, Shinji Sawano, Aleksi Lehtonen, Shigehiro Ishizuka

**Affiliations:** 1 Kyushu Research Center, Forestry and Forest Products Research Institute (FFPRI), Kumamoto-city, Kumamoto, Japan; 2 Department of Forest Soils, FFPRI, Tsukuba, Ibaraki, Japan; 3 Graduate School of Agricultural and Life Sciences, The University of Tokyo, Bunkyo-ku, Tokyo, Japan; 4 Department of Disaster Prevention, Meteorology and Hydrology, FFPRI, Tsukuba, Ibaraki, Japan; 5 River Basin Research Center, Gifu University, Gifu-city, Gifu, Japan; 6 Hokkaido Research Center, FFPRI, Sapporo, Hokkaido, Japan; 7 Natural Resources Institute Finland, Helsinki, Finland; West Virginia University, UNITED STATES

## Abstract

Spatiotemporal prediction of the response of planted forests to a changing climate is increasingly important for the sustainable management of forest ecosystems. In this study, we present a methodology for estimating spatially varying productivity in a planted forest and changes in productivity with a changing climate in Japan, with a focus on Japanese cedar (*Cryptomeria japonica D*. *Don*) as a representative tree species of this region. The process-based model Biome-BGC was parameterized using a plant trait database for Japanese cedar and a Bayesian optimization scheme. To compare productivity under historical (1996–2000) and future (2096–2100) climatic conditions, the climate scenarios of two representative concentration pathways (i.e., RCP2.6 and RCP8.5) were used in five global climate models (GCMs) with approximately 1-km resolution. The seasonality of modeled fluxes, namely gross primary production, ecosystem respiration, net ecosystem exchange, and soil respiration, improved after two steps of parameterization. The estimated net primary production (NPP) of stands aged 36–40 years under the historical climatic conditions of the five GCMs was 0.77 ± 0.10 kgC m^-2^ year^-1^ (mean ± standard deviation), in accordance with the geographical distribution of forest NPP estimated in previous studies. Under the RCP2.6 and RCP8.5 scenarios, the mean NPP of the five GCMs increased by 0.04 ± 0.07 and 0.14 ± 0.11 kgC m^-2^ year^-1^, respectively. The increases in annual NPP were small in the southwestern region because of the decreases in summer NPP and the small increases in winter NPP under the RCP2.6 and RCP8.5 scenarios, respectively. Under the RCP2.6 scenario, Japanese cedar was at risk in the southwestern region, in accordance with previous studies, and monitoring and silvicultural practices should be modified accordingly.

## Introduction

At present, spatiotemporal prediction of the response of planted forests to a changing climate is increasing in importance. The planted forest area worldwide reached 293.9 million ha, which is 7% of total forest area, by 2020 [1]. This increase has highlighted the importance of predicting the spatial responses of planted forests to changing climatic conditions in terms of material and energy flows in terrestrial ecosystems. In particular, predicting the future risk of decreasing productivity and carbon (C) sequestration in planted forests and modeling of C dynamics [2] are expected play important roles.

Prediction of a planted forest’s response to climate change through modeling of C dynamics depends strongly on the climate scenarios employed. To fully use the information in climate scenarios and quantify changes in the stock and flow of C, process-based modeling, which quantifies processes such as photosynthesis and respiration and represents them as numerical expressions, is an effective approach. Several process-based models, including ORCHIDEE [3], LPJ-GUESS [4] and 3-PG [5], have been applied to planted forests, and Biome-BGC (BBGC) has been used to model various tree species in Europe [6, 7] and East Asia [8, 9].

Appropriate parameterization is an essential step in the use of process-based models. Process-based models have been parameterized for various plant functional types [10]. In recent years, however, a powerful dataset of plant traits has been developed for each tree species [11, 12], enabling species-specific or plant trait-based parameterization of process-based models [13] with a focus on the relationship between the diversity of plant traits and the ecosystem response to climate change [14]. A plant trait database (PTDB) for Japanese cedar (*Cryptomeria japonica D*. *Don*), which is described later, and Japanese cypress (*Chamaecyparis obtusa*) was published recently [15]. We can refer to this PTDB for representative values of eco-physiological parameters to apply in process-based models, such as leaf nitrogen (N) content and specific leaf area (SLA). Thus, the PTDB enables model users to select parameters in a physiologically supported manner. In addition, Bayesian calibration [16, 17] or optimization (BO) is an effective approach for certain parameters with little or no data in the PTDB. These Bayesian approaches support the selection of parameters through repeated model runs, known as Markov chain Monte Carlo (MCMC) sampling, in which data are effectively used to estimate optimal parameter values and their uncertainties [16, 17].

The modeling research described above has also focused on the coastal region of East Asia, which is the region with the highest proportion of planted forest [1] and is characterized by a relatively warm and humid climate. Japan has the unique characteristic that a single tree species is planted across a wide climatic range, from subtropical to cool temperate [18]. Thus, the parameterization and validation of process-based models for planted forests in this region remain a challenge, and clarification of the application range of such models is needed. On the other hand, Japan increased its stock of planted trees at an extremely high rate after World War II [19]. Forest covers two-thirds of the land area at present, and a stand age of approximately 50–60 years is common for planted forests [20]; those stands are now mature and can be harvested. Thus, forest resources in Japan are currently rich. However, the effects of ongoing climate change on mature tree plantations remain unclear, and previous research has suggested the potential risk of declining stand productivity [5, 21, 22]. Therefore, demand for spatiotemporal prediction of the productivity of planted forests in Japan is increasing to support the implementation of adaptation strategies for climate change. Research into the productivity of Japanese cedar plantations, which cover 18% of the total forested area and 44% of the planted forest area in Japan [20], must be undertaken with the highest priority due to its importance to the timber industry.

Previous studies have shown that Japanese cedar may be vulnerable to climate change, especially drier climatic conditions. Matsumoto et al. [22] estimated the vulnerability of Japanese cedar to a warming climate at the national scale based on two indices—namely, the ratio of annual transpiration to precipitation and soil water-holding capacity. They found that the area of high vulnerability is distributed in the western part of Japan and that Japanese cedar growth may decrease extensively from western to eastern Japan under future climate scenarios with increases in mean annual temperature of 2.2–3.2 degrees C by 2081–2100 [22]. Matsumoto et al. [22] calculated transpiration [23] based on stomatal conductance [24–26] and vapor pressure deficit (VPD) using second mesh order (approximately 10-km resolution) climate scenarios at a monthly timescale. At present, third mesh order (approximately 1-km resolution) climate scenarios at a daily timescale are available [27], and prediction of the stomatal response of Japanese cedar to climate scenarios with high spatiotemporal resolution can be undertaken. In addition, quantitative prediction of the changes in stand productivity is essential for the development of forestry policy.

Based on this background, we focus on estimating changes in stand productivity, evaluated as net primary production (NPP), rather than on mortality under severe climatic conditions. In extremely severe climate scenarios, increased mortality may be caused by hydraulic failure and reduced production of non-structural C [28]; process-based models covering these processes are being developed rapidly and will become popular in the near future. In this study, our general objective is to contribute to the development of adaptation strategies for climate change for the forestry sector in East Asia. As a specific objective, we parameterize a process-based model for planted forests in Japan at the national scale to quantify the climatic limitations on stand productivity and detect regions with possible declines in NPP, with a focus on Japanese cedar as a representative tree species that grows in a warm and humid region of East Asia.

## Materials and methods

### Biome-BGC (BBGC)

We used the process-based ecosystem model BBGC ver.4.2 [29] as the basis for modeling C dynamics. BBGC is a two-leaf model that simulates the stocks and fluxes of C, N, water and energy. This model requires input data on daily maximum, minimum, and mean air temperatures; precipitation; daytime VPD and solar radiation; and day length. BBGC also requires eco-physiological data (see section *Parameterization*) and site data (see section *Site data*) to run its simulations. The NPP is calculated by gross primary production (GPP) minus autotrophic respiration in daily routine. GPP is calculated using Farquhar’s model [24] connected with the stomatal conductance model [30]. In this routine, the function of the maximum rate of carboxylation (*V*_*cmax*_) is associated with the parameterization conducted in this study, as follows:

$$V_{cmax}=\frac{FLNR\times 7.16\times ACT}{CN\_leaves\times SLA},$$ (1)

where *FLNR* is the fraction of leaf N in Rubisco, *ACT* is the activity of Rubisco scaled based on temperature and the concentrations of oxygen (O_2_) and carbon dioxide (CO_2_), and *CN_leaves* is the C:N ratio in leaves. The photosynthesis rate is reduced with four scaling factors—namely, air temperature, VPD (S1 Fig in S1 File), soil water potential (S1 Fig in S1 File), and solar radiation. Autotrophic respiration comprises maintenance respiration, which is a function of temperature and the N pool of the living tree, and growth respiration, which is a fixed proportion of GPP. The allocation of C to tree organs, such as leaves, stems, coarse roots, and fine roots, is determined as a fixed ratio based on eco-physiological parameters. Other processes such as the decomposition of woody debris and soil organic matter have been described in detail previously [29]. In this study, we use BBGC as applied in many previous studies of planted forests to estimate changes in NPP based on a simple physiological response without acclimation to climatic factors, i.e., increased temperature and elevated CO_2_ concentration.

First, we made minor changes to the BBGC code. The turnover rate of fine roots was separated from that of leaves, as in previous studies [6, 7, 31]. In addition, the function driving the soil water retention curve, which represents the relationship between soil water content and soil matric potential, was replaced; the original pedo-transfer function based on soil texture (i.e., sand, silt, and clay content) was switched to the function of van Genuchten’s model [32]. This change affected the estimation of GPP, in combination with site data (see section *Soil water retention curve*), due to the reduction function for stomatal conductance associated with soil water potential (S1 Fig in S1 File).

### Plant trait database (PTDB)

We used a PTDB for Japanese cedar [15] for the parameterization of BBGC. The PTDB contains 16,410 data entries for 177 types of plant traits in Japanese cedar, which were collected through review of previous studies published since 1950. The PTDB was also used for the validation of modeled NPP through extraction of observed NPP values from the database (see section *Statistics and validation*).

### Flux data for Japanese cedar plantations

In addition to the PTDB, monitored and calculated flux values for Japanese cedar at two eddy covariance monitoring sites, the Takayama coniferous forest (TKC) and Kahoku experimental watershed (KHW), were used for the parameterization of BBGC.

#### Takayama coniferous forest (TKC)

**Fig 1:**
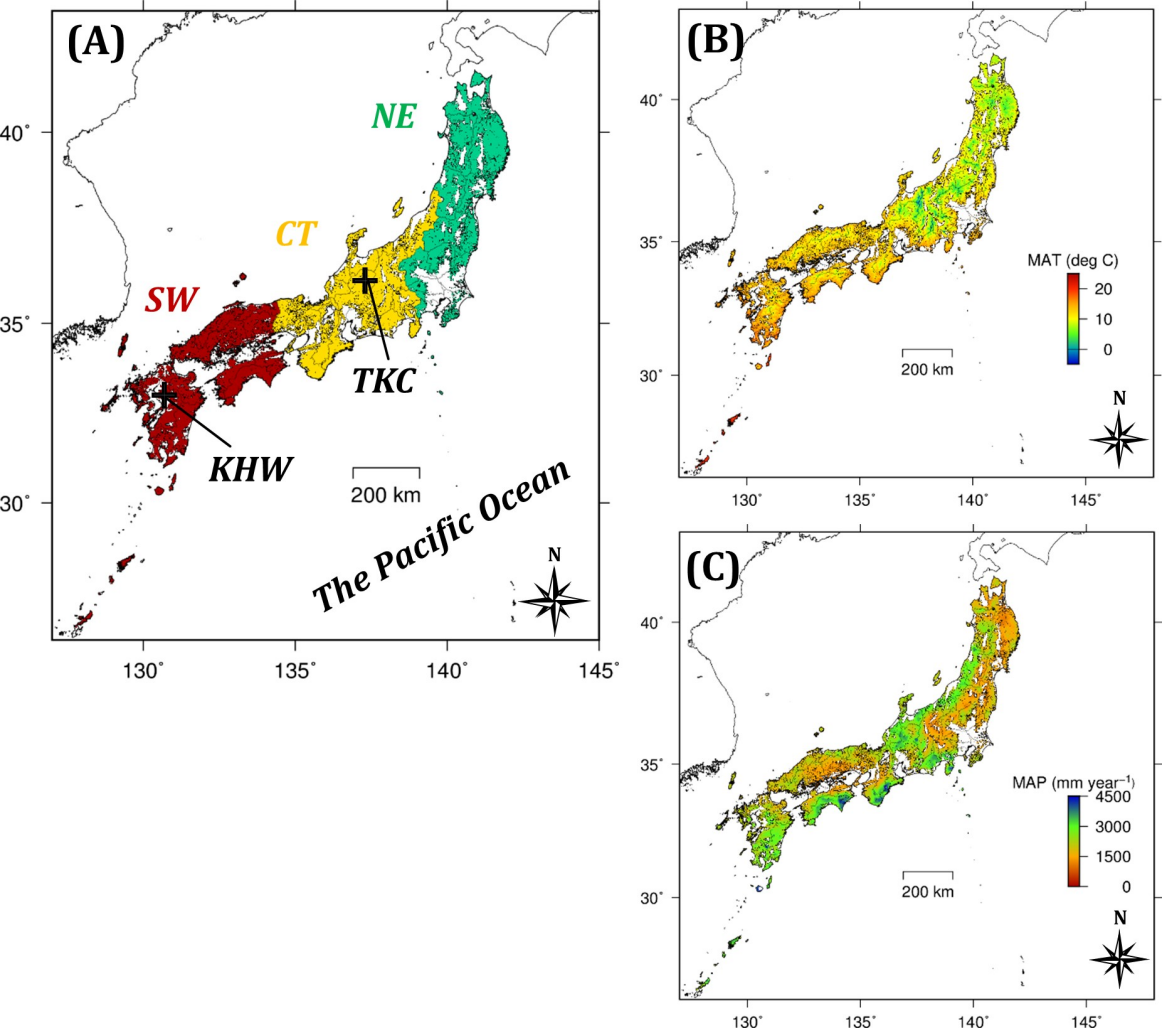
Maps of regional blocks, flux-monitoring sites, and climate in Japan. (A) Locations of the three blocks and two flux-monitoring sites, and (B) mean annual temperature (MAT) and (C) mean annual precipitation (MAP) in the 1996–2000 period. Red, yellow, and green in map (A) indicate the southwestern (SW), central (CT), and northeastern (NE) blocks, respectively. Maps (B) and (C) were constructed using historical data from MIROC5 (Model for Interdisciplinary Research On Climate Version Five).

The TKC is located in a cool temperate region (36.140 deg N, 137.371 deg E; Fig 1A). The tree plantation was established in 1961 or later. The mean annual temperature is 10.7 deg C (from 1967 to 2006) and the mean annual precipitation is 1722 mm (from 1967 to 2006). The plant area index ranges from 4.9 to 5.2 m^2^ m^-2^. Further site information is available from the Asiaflux website (http://asiaflux.net/index.php?page_id=111). The monitoring data used in this study include daily datasets of climate, GPP, ecosystem respiration (RE), and net ecosystem change (NEE) estimated using the eddy covariance method for 2006 (stand age of 46 years). For more detailed information about the flux calculation and gap-filling processes, see Saitoh et al. [33]. Climatic input data for BBGC over 45 years (1961–2005) were obtained through correction of data from the nearest meteorological station.

#### Kahoku experimental watershed (KHW)

The KHW is located in a warm temperate region (33.137 deg N, 130.710 deg E; Fig 1A). The tree plantation was established in 1956. The mean annual temperature is 15.1 deg C (from 2000 to 2003) and the mean annual precipitation is 2106 mm (from 2001 to 2003). The leaf area index as measured with a LAI-2000 plant canopy analyzer (Li-Cor, Inc., Lincoln, NE) ranges from 3.6 to 5.2 m^2^ m^-2^. Further information is available from the FFPRI FluxNet Database website [34]. The monitoring data used in this study were daily datasets of climate (2000–2003) and NEE (2001–2003, stand age of 46–48 years). Because Japanese cypress grows in part of the watershed, we extracted flux mainly from the Japanese cedar stand based on wind direction (from the right side of the site), as described by Shimizu et al. [35]. Then, we conducted gap-filling through the mutual imputation method (NORM software) using *in-situ* measurements of meteorological factors (solar radiation, net radiation, air temperature, VPD, and wind speed; [35, 36]) and generated daily NEE for the Japanese cedar stand. As in the TKC, NEE in the KHW was separated into GPP and RE. We averaged daily GPP to generate monthly data and used these data for parameterization, as the calculated response of Japanese cedar to climate in the KHW was considered less reliable at the daily timescale according to the gap-filling procedure described above. In addition to monthly GPP, RE, and NEE, monthly soil respiration measurements were up-scaled using soil temperature and the site-specific regression model [37]. Climatic input data for BBGC over 21 years (1978–1999) were obtained and corrected using data from the nearest meteorological station.

### Parameterization

We varied 12 of 44 eco-physiological parameters in BBGC in two steps, while considering the balance between simplicity and effectiveness of parameterization. STEP-0 uses the parameter set prepared for evergreen needle-leaf forest in BBGC ver.4.2 (Table 1). In STEP-1 and -2, we mainly updated the eco-physiological parameters of leaves, which are the basis of the two-leaf model BBGC and control photosynthesis at the daily timescale. On the other hand, the eco-physiological parameters of turnover, except for fine roots and the chemical composition of tree organs, which control processes at the monthly or annual timescales, remained unchanged from STEP-0 in this study, mainly due to sparse data in the PTDB. In addition, parameters related to C allocation, the mechanism of which remains unclear for Japanese cedar, were also unchanged.

**Table 1:** Eco-physiological parameters used in BBGC.

No	Eco-physiological parameter	Unit	STEP-0	STEP-2	Note
1	1 = WOODY 0 = NON-WOODY	(flag)	1		
2	1 = EVERGREEN 0 = DECIDUOUS	(flag)	1		
3	1 = C3 PSN 0 = C4 PSN	(flag)	1		
4	1 = MODEL PHENOLOGY 0 = USER-SPECIFIED PHENOLOGY	(flag)	1		
5	yearday to start new growth (when phenology flag = 0)	(day of year)	0		
6	yearday to end litterfall (when phenology flag = 0)	(day of year)	0		
7	transfer growth period as fraction of growing season	(prop.)	0.3		
8	litterfall as fraction of growing season	(prop.)	0.3		
9	annual leaf turnover fraction	(year^-1^)	0.25		
10	annual fine root turnover fraction	(year^-1^)	0.25	**0.69**	STEP-1, Noguchi *et al*. 2007
11	annual live wood turnover fraction	(year^-1^)	0.7	** **	
12	annual whole-plant mortality fraction	(year^-1^)	0.005	** **	
13	annual fire mortality fraction	(year^-1^)	0.005	**0**	STEP-1, statistics on forest fire in Japan
14	(ALLOCATION) new fine root C : new leaf C	(ratio)	1	** **	
15	(ALLOCATION) new stem C : new leaf C	(ratio)	2.2	** **	
16	(ALLOCATION) new live wood C : new total wood C	(ratio)	0.1	** **	
17	(ALLOCATION) new croot C : new stem C	(ratio)	0.3	** **	
18	(ALLOCATION) current growth proportion	(prop.)	0.5	** **	
19	C:N of leaves	(gC gN^-1^)	42	**37**	STEP-1, median in PTDB (N = 1731)
20	C:N of leaf litter, after retranslocation	(gC gN^-1^)	93	**83**	STEP-1, scaling by C:N of leaves
21	C:N of fine roots	(gC gN^-1^)	42	**70**	STEP-1, median in PTDB (N = 39)
22	C:N of live wood	(gC gN^-1^)	50	**55**	STEP-1, scaling by C:N of dead wood
23	C:N of dead wood	(gC gN^-1^)	729	**801**	STEP-1, median in PTDB (N = 69)
24	leaf litter labile proportion	(DIM)	0.32	** **	
25	leaf litter cellulose proportion	(DIM)	0.44	** **	
26	leaf litter lignin proportion	(DIM)	0.24	** **	
27	fine root labile proportion	(DIM)	0.3	** **	
28	fine root cellulose proportion	(DIM)	0.45	** **	
29	fine root lignin proportion	(DIM)	0.25	** **	
30	dead wood cellulose proportion	(DIM)	0.76	** **	
31	dead wood lignin proportion	(DIM)	0.24	** **	
32	canopy water interception coefficient	(LAI^-1^ day^-1^)	0.041	** **	
33	canopy light extinction coefficient	(DIM)	0.5	** **	
34	all-sided to projected leaf area ratio	(DIM)	2.6	** **	
35	canopy average specific leaf area (projected area basis)	(m^2^ kgC^-1^)	12	**10.6**	STEP-1, median in PTDB (N = 240)
36	ratio of shaded SLA:sunlit SLA	(DIM)	2	** **	
37	fraction of leaf N in Rubisco	(DIM)	0.04	**0.0152**	STEP-2, Bayesian optimization
38	maximum stomatal conductance (projected area basis)	(m s^-1^)	0.003	**0.0031**	STEP-1, median in PTDB (N = 57)
39	cuticular conductance (projected area basis)	(m s^-1^)	0.00001	** **	
40	boundary layer conductance (projected area basis)	(m s^-1^)	0.08	** **	
41	leaf water potential: start of conductance reduction	(MPa)	-0.6	** **	
42	leaf water potential: complete conductance reduction	(MPa)	-2.3	** **	
43	vapor pressure deficit: start of conductance reduction	(Pa)	930	**135**	STEP-2, Bayesian optimization
44	vapor pressure deficit: complete conductance reduction	(Pa)	4100	**1825**	STEP-2, Bayesian optimization

#### STEP-1

Nine eco-physiological parameters were varied in STEP-1. The annual fire mortality was changed from 0.005 to 0. We assumed that the risk of forest fire in Japan is quite low relative to other countries based on the long-term trend of forest fires [38]. In particular, no fire history has been recorded at the TKC or KHW. Then, the turnover of fine roots was changed from 0.25 to 0.69 year^-1^ (value of Konôpka cited in Noguchi et al. [39]), considering its effect on biomass accumulation [31].

We also changed five parameters related to the C:N ratio, SLA, and Max_sc (Table 1). These parameters are sensitive to NPP [40] and have relatively rich data in the PTDB. We used median values in the PTDB, as some parameters were ratios or exhibited log-normal distributions in the PTDB (S2 Fig in S1 File). The C:N ratios of leaf litter after retranslocation and of live wood were scaled to those of leaves and dead wood.

#### STEP-2

Three additional eco-physiological parameters were optimized in STEP-2. We applied BO using the algorithms employed for Bayesian calibration [16] with slight modification. In BO, the likelihood function p (*E*) was defined for each site, the TKC and KHW, as follows:

$$p(E=D-M(\theta ))=\prod_{i=1}^{n}{\varphi (D_{i}-M_{i}(\theta );0,(0.2D_{ave})}^{2}),$$ (2)

where *E* is the difference between the observed data, *D*, and output of BBGC, *M*, based on the vector of three eco-physiological parameters, *θ*. The term *i* is an index of the observed data and the output of BBGC, *n* is the number of observational data, *φ* denotes a Gaussian probability density function with a given mean and variance, and *D*_*ave*_ is the average *D* for the TKC or KHW. Then, we combined the p(*E*) of the TKC and KHW and estimated a vector for the parameters that provide maximum likelihood. As described below, we constrained the model using GPP at the TKC and KHW simultaneously and weighted it using two likelihood functions to avoid being affected more strongly by the TKC, for which we have more data than for the KHW. The Bayesian calibration procedure was described in detail by Van Oijen et al. [16].

GPP in the TKC (daily data in 2006, 365 count) and KHW (monthly data in 2001–2003, 36 count) were used as constraints on BO. In this study, we focused on the improvement of modeled GPP as a C input to the ecosystem, and only validated the modeled RE (also, soil respiration in the KHW) and NEE values through comparisons with calculated RE and observed NEE, respectively. As RE is related to the decomposition process of organic matter over several decades and is affected by many eco-physiological parameters for which little information is available, we found that optimization using RE data complicated the procedure of parameterization, even when the effective approach of a hierarchal parameterization scheme was used [41].

We optimized the fraction of leaf N in Rubisco (FLNR) and VPD values for starting and ending the reduction of stomatal conductance (VPD_rsc_start and VPD_rsc_end, respectively). Although the PTDB contains little or no data for these parameters, we consider these parameters essential for the improvement of modeled GPP. The prior distribution in BO was uniform, and the range of FLNR was 25–200% of 0.04 (0.01–0.08), the value in STEP-0; those of VPD_rsc_start and VPD_rsc_end were 10–100% of 930 Pa (93–930 Pa) and 25–100% of 4100 Pa (1025–4100 Pa), respectively. We set these ranges of VPD_rsc_start and VPD_rsc_end for BO based on a previous report stating that canopy conductance of Japanese cedar can decrease when VPD is much lower than 1.0 kPa [42]. In MCMC sampling for BO, three chains of 100,000 iterations were calculated, starting from the 25^th^, 50^th^, and 75^th^ percentiles of the prior distribution.

### Site data

We prepared site data as an *ini* file in BBGC. The mesh for calculation contains forest areas larger than 0.4 km^2^ (approximately 40% of the total area in the mesh) based on information in the National Forest Resources Database ([43], compiled at the third mesh order). The mesh for Hokkaido, the northernmost prefecture of Japan, was excluded in this study because plantations of Japanese cedar are very scarce in that region, except for a small proportion of the southern area, and because few observational data of NPP and biomass are available for comparison with model outputs. We set shortwave albedo to a uniform value of 0.15 in the *ini* file.

#### Soil thickness

We prepared a map of the thicknesses of the A and B soil horizons and used their sum as soil thickness in the *ini* file in BBGC (S3 Fig in S1 File). The data source is soil profiles at 2056 points in Japan, obtained from National Forest Soil Carbon Inventory Project [44] and a legacy dataset compiled by Morisada et al. [45]. We first extracted the maximum soil thickness within the area represented as a coarse mesh of 15-km resolution with regional-scale values of soil thickness (*N* = 671). The map of soil thickness at the third mesh order with 1-km resolution was estimated through ordinary kriging from regional-scale data using the Geostatistical Analyst Extension of ArcGIS Pro 10.5 software (ESRI Inc., USA). Thus, the interpolated map shows the maximum potential soil thickness at the regional scale (*ca*. 15 km) in Japanese forests.

#### Soil water retention curve

We prepared the parameters related to the soil water retention curve of van Genuchten ([32]; called vG parameters in this study) for each soil type based on the current forest soil classification system (FSD system) for Japan [46]. The equation of van Genuchten is as follows:

$$\theta (\psi )=(\theta_{s}-\theta_{r}){\left[\frac{1}{1+{\left(\alpha \psi \right)}^{n}}\right]}^{m}+\theta_{r},$$ (3)

where *θ*(*ψ*) is the volumetric soil water content (m^3^ m^-3^) as a function of *ψ*, the soil matric potential (MPa). *θ*_s_ and *θ*_r_ are the saturated and residual soil water contents in volume, respectively, and *α*, and *n*, and *m* (= 1 − 1/*n*) are curve-fitting parameters. We first converted the vector soil map at a scale of 1:200,000 [47] into a raster soil map of the third mesh order (approximately 1-km resolution) based on the soil type with maximum coverage. The soil type in the map of the National Land Agency, which differs from that of the FSD system, was converted to align with the FSD system using the criteria proposed by Morisada et al. [45] to allocate the representative vG parameters of the A and B horizons to the map. Representative vG parameters of the A and B horizons of eight soil types were obtained through fitting of the vG function to soil water retention curve datasets from previous studies, which were allocated to 13 soil types (S4 Table in S1 File). Then, the vG parameters of the A and B horizons were weight-averaged based on soil thickness. To express the soil water content of the entire soil profile at a certain value of soil matric potential, arithmetic and geometric averaging [48] were used for *θ*_s_ and *θ*_r_, and for *α* and *n*, respectively. The map of effective soil water capacity in the soil profile, calculated based on soil thickness, *θ*_s_ and *θ*_r_, is shown in S3 Fig in S1 File.

#### Deposition and biological fixation of nitrogen

In this study, differences in the input of N among grids were expressed based on differences in the deposition of N (S3 Fig in S1 File). We obtained the sum of dry and wet deposition of N in a 15-km grid in 2012 from data simulated using an atmospheric chemical transport model [49], which was used in Nishina et al. [50]. Deposition data at the third mesh order with 1-km resolution were interpolated through ordinary kriging.

On the other hand, no spatial information on the biotic fixation of N was available, although it occurs in the litter layer of Japanese cedar plantations [51, 52]. Therefore, we assigned a relatively low constant rate of biotic N fixation across all regions (0.40 gN m^-2^ year^-1^) considering the ranges associated with lichen (0–0.20 gN m^-2^ year^-1^), bryophytes (0.07–1.00 gN m^-2^ year^-1^), and litter (0.10–0.20 gN m^-2^ year^-1^) in temperate forests [53].

### Spin-up and normal run procedures

In spin-up runs for the TKC and KHW sites, we used the eco-physiological parameters of STEP-0, set the atmospheric CO_2_ concentration to the value for the first year of climate data, and fixed the N deposition rate to 0.1 gN m^-2^ year^-1^. To determine the initial conditions of the normal runs for the TKC and KHW, we cut 100% of the biomass C in existing forest generated during the spin-up run, removed above-ground biomass C, and increased leaf C by 0.1 kgC m^-2^. In the normal runs for the TKC and KHW sites, we set the N deposition rate to 1.1 gN m^-2^ year^-1^. For the TKC, climate data for 39 years before 2000 (1961–1999) were used in the spin-up run and those for 46 years (1961–2006) were used in the normal run. For the KHW, climate data for 21 years before 2000 (1978–1999) were used in the spin-up run, those for 26 years (1978–2003) were used in the normal run, and climate data for the 1978–1999 period were applied to the young stand (1956–1977).

For the initial conditions of the normal run at the national scale, we set the cutting (and removal) ratio of biomass C in the existing forest to 90%. The cutting ratio has been controlled in previous studies to explore the effect of forest disturbance on simulated flux [9, 29]. This ratio was determined practically in this study based on preliminary analysis, and a cutting ratio lower than 100% improved the stability of the calculation through increased leaf biomass at the initial growth stage, especially in severely cold regions, although the percentage of such regions in the total mesh area is quite small. We confirmed that changing the cutting ratio of biomass C in existing forest scarcely affected the modeled NPP and increased rate of vegetation biomass, although the baseline level of vegetation biomass increased (see section of Result, *Recorded and modeled NPP and biomass C relative to stand age*). Other forest management options, such as thinning, were not considered in the normal run because they were too spatially diverse to capture with 1-km resolution and were outside of the objectives of this study.

### Climate scenarios

We used climate scenarios that were statistically downscaled, i.e., Inverse Distance Weighted method, by NARO [27] (Nishimori et al., 2019; NAROv2.7r). The original dataset is the product of phase 5 of the Coupled Model Intercomparison Project (CMIP5). These scenarios were generated using five global climate models (GCMs; Table 2) under two representative concentration pathways, RCP2.6 and RCP8.5, following the conventions of the Intergovernmental Panel on Climate Change [54]. RCP2.6 is a representative scenario based on low emissions of greenhouse gasses (GHGs) that aims to keep global warming below 2 degrees C above pre-industrial temperatures [54]. Meanwhile, RCP8.5 is based on very high GHG emissions, higher than those in scenarios without additional efforts to constrain GHG emissions (baseline scenarios). The downscaled dataset contains climate scenarios based on historical trends (1981–2005) and future predictions (2006–2100) [27]. The atmospheric CO_2_ concentration for each RCP is shared among GCMs and was obtained from the RCP Database [55, 56]. The atmospheric CO_2_ concentration in 2000 was 368.9 ppm and those in 2100 under RCP2.6 and RCP8.5 are 420.9 and 935.9 ppm, respectively.

**Table 2:** The five GCMs used for climate prediction in this study.

GCM	Institute	Country
HadGEM2-ES	Met Office Hadley Centre	UK
MRI-CGCM3	Meteorological Research Institute	Japan
CSIRO-Mk3-6-0	Commonwealth Scientific and Industrial Research Organisation	Australia
MIROC5	The university of Tokyo, National Institute for Environmental Studies and Japan Agency for Marine-Earth Science and Technology	Japan
GFDL-CM3	Geophysical Fluid Dynamics Laboratory, National Oceanic and Atmospheric Administration	United States

In BBGC, some climate input data are based on daytime-average values rather than daily-average values. Therefore, we used daily maximum and minimum air temperatures (Tmax and Tmin, deg C) and precipitation (mm day^-1^) in the climate scenarios in this study, and employed MT-CLIM module ver.4.3 [57, 58] to generate the remaining input data for BBGC, i.e., daily mean air temperature (deg C), daytime VPD (Pa), solar radiation (W m^-2^), and day length (sec). In our exploratory analysis, the spatiotemporal trend of daytime VPD from the MT-CLIM module was similar to that of daily VPD estimated from daily relative humidity in the climate scenarios of MIROC5 (Model for Interdisciplinary Research On Climate Version Five), with both VPDs increasing in summer, especially in the western part of Japan.

**Fig 2:**
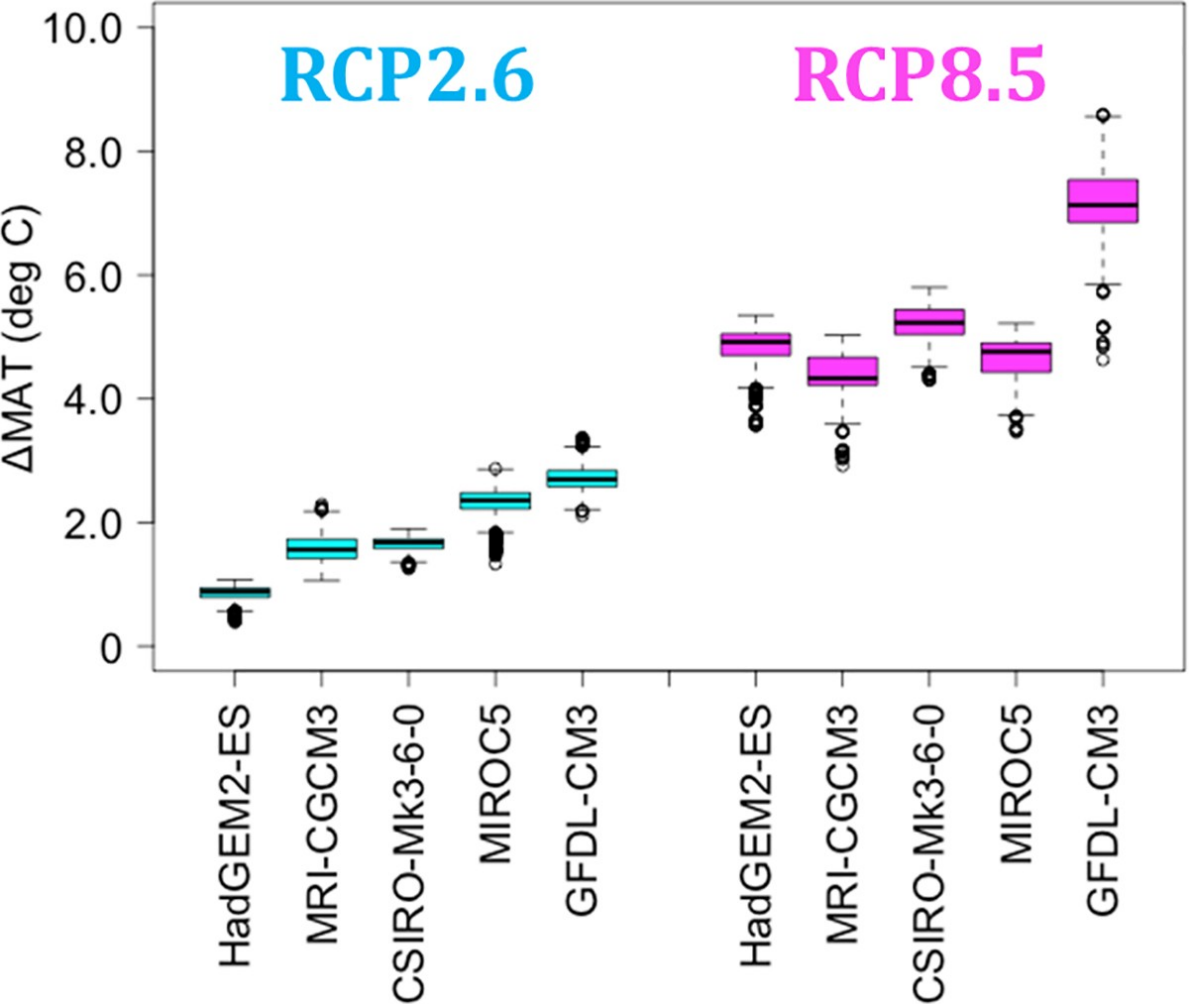
Trends in ΔMAT according to five GCMs. Comparison between H_2000 and F_2100.

Here, we mainly summarize the characteristics of the temperature increases according to five GCMs for the target area (see section *Target stand age and area for prediction*). In this study, we considered the outputs for 1996–2000 as the historical trend (H_2000) and those for 2096–2100 as future predictions (F_2100). The mean annual temperature (MAT) for forested areas from the five GCMs for H_2000 was 11.3 deg C, with high values in the southwestern region (Table 3, Fig 1B) and little variation among the GCMs. The increases in MAT (ΔMAT) from H_2000 to F_2100 were 1.8 and 5.3 deg C for RCP2.6 and RCP8.5, respectively, averaged over the five GCMs (Table 3). The ΔMAT from MIROC5, which is well-documented in this study, was the second highest for RCP2.6 and fourth highest for RCP8.5 (Fig 2). The mean annual precipitation (MAP) from the five GCMs for H_2000 was 2127 mm year^-1^ (Table 3, Fig 1C) and increased along with MAT in future scenarios. The mean ΔMAP in MIROC5 was relatively low for RCP2.6 and high for RCP8.5 relative to those of the other GCMs (Table 3). The increasing trends for daytime VPD from the five GCMs, calculated using MT-CLIM, were similar to those for MAT, and are shown in S5 Table in S1 File along with the trends for solar radiation.

**Table 3:** MAT and MAP and their changes in future scenarios.

		MAT in H_2000			ΔMAT in F_2100						MAP in H_2000			ΔMAP in F_2100					
GCM	Block				RCP2.6			RCP8.5							RCP2.6		RCP8.5		
					(deg C)									(mm year^-1^)					
5GCMs	SW	13.8	±	2.4	1.8	±	0.7	4.9	±	1.0	2280	±	625	337	±	312	438	±	292
	CT	10.9	±	3.2	1.8	±	0.6	5.3	±	1.0	2315	±	639	259	±	255	310	±	194
	NE	9.1	±	2.3	1.9	±	0.7	5.6	±	1.2	1764	±	434	271	±	162	354	±	230
	Total	11.3	±	3.3	1.8	±	0.7	5.3	±	1.1	2127	±	628	288	±	254	367	±	248
MIROC5	SW	13.6	±	2.4	2.5	±	0.2	4.3	±	0.2	2241	±	585	9	±	87	713	±	271
	CT	10.8	±	3.2	2.4	±	0.1	4.7	±	0.2	2343	±	619	50	±	106	459	±	159
	NE	9.1	±	2.2	2.1	±	0.2	4.9	±	0.1	1831	±	424	229	±	127	502	±	157
	Total	11.2	±	3.2	2.3	±	0.3	4.7	±	0.3	2145	±	594	94	±	143	557	±	231

Mean ± s.d.; SW, CT, and NE indicate the southwestern, central, and northeastern blocks, respectively.

### Target stand age and area for prediction

We estimated the average NPP for 36–40-year-old stands to reduce yearly fluctuations caused by climate data, as the age of 40 years is commonly used as a site index in Japan. In the calculation for H_2000 (1996–2000 for 36–40-year-old stands), climate data for 1981–2000 were used twice to simulate a 40-year-old stand, whereas in the calculation for F_2100 (2096–2100 for 36–40-year-old stands), climate data for 2061–2100 were used once, except in HadGEM2-ES, which contains climate data for 2060–2099. The ΔNPP of each point of mesh was calculated as follows:

$$\Delta NPP=NPP_{F\_2100}-NPP_{H\_2000},$$ (4)

where NPP_F_2100_ and NPP_H_2000_ are mean NPP for F_2100 and H_2000, respectively. In total, 196,928 points of third mesh order were used for prediction (BBGC_full), covering all of Japan except Hokkaido. We divided this area into three blocks (Fig 1A)—namely, the southwestern (SW; 33% of total forest area, covering the Kyushu, Okinawa, Chugoku, and Shikoku Japanese administrative districts), central (CT; 35%, Kinki, Tokai, and Hokuriku districts), and northeastern (NE, 32%, Kanto and Tohoku districts) blocks. In addition, we used a dataset of 1967 points (BBGC_sample) that were systematically sampled from BBGC_full to analyze the differences between relatively warm (May to October) and cool (November to April) seasons, those among the five GCMs, and the effects of climate factors on GPP and NPP. We confirmed that the probabilistic distribution of NPP in BBGC_sample was approximately the same as that in BBGC_full (S6 Fig in S1 File), indicating that the use of BBGC_sample had little impact on the results and conclusions of this study.

### Statistics and validation

The normalized root mean square error (NRMSE) for the model output based on eco-physiological parameters in STEP-0, -1, and -2 was calculated as follows:

$$NRMSE=\frac{\sqrt{\frac{1}{n}\sum_{i=1}^{n}{\left(D_{i}-M_{i}\right)}^{2}}}{{(D}_{max}-D_{min})},$$ (5)

where, as in Eq (2), *D* and *M* are the observed value and modeled output from BBGC, respectively. The term *i* is an index of the observed data and the output of BBGC, and *n* is the number of observations. *D*_max_ and *D*_min_ are the maximum and minimum observed values, respectively, across the TKC or KHW datasets.

The NPP of planted forests, not only for Japanese cedar but in general, can be affected by growth stage [59]. Therefore, we validated the stand age used for estimating NPP, i.e., 36–40 years, by extending the stand age from 40 to 60 years in H_2000 using climate data from MIROC5 for 1981–2000 three times. We compared the trend in mean NPP with age between the model output and observations. We extracted observed NPP values from the PTDB [15] by filtering for a stand age of 31–50 years. We also analyzed the increases in biomass C with age and extracted wood volume data from the National Forest Inventory (NFI) for 2009–2013 by filtering for a stand age of 30–60 years without thinning history. Then, we converted the volume to biomass C using a biomass expansion factor (BEF; [60]). In addition, as one step in the validation of the estimated range of NPP, we compared the NPP from H_2000 in STEP-2 between cedar-dominated and non-cedar-dominated mesh points based on data in the National Forest Resources Database [43] (see S7 Fig in S1 File), although the target of this study is the total forested area in Japan. This comparison was undertaken based on the idea that cedar-dominated areas are currently under environmental conditions that favor the growth of Japanese cedar plantations relative to those of non-cedar-dominated areas.

In this study, we did not conduct uncertainty analysis, which would require the probabilistic density of all parameters but instead analyzed the sensitivity of modeled NPP to selected eco-physiological and site parameters. The result of sensitivity analysis is described in supporting information files (S8 Text in S1 File).

## Results

### Simulated flux before and after parameterization

**Fig 3:**
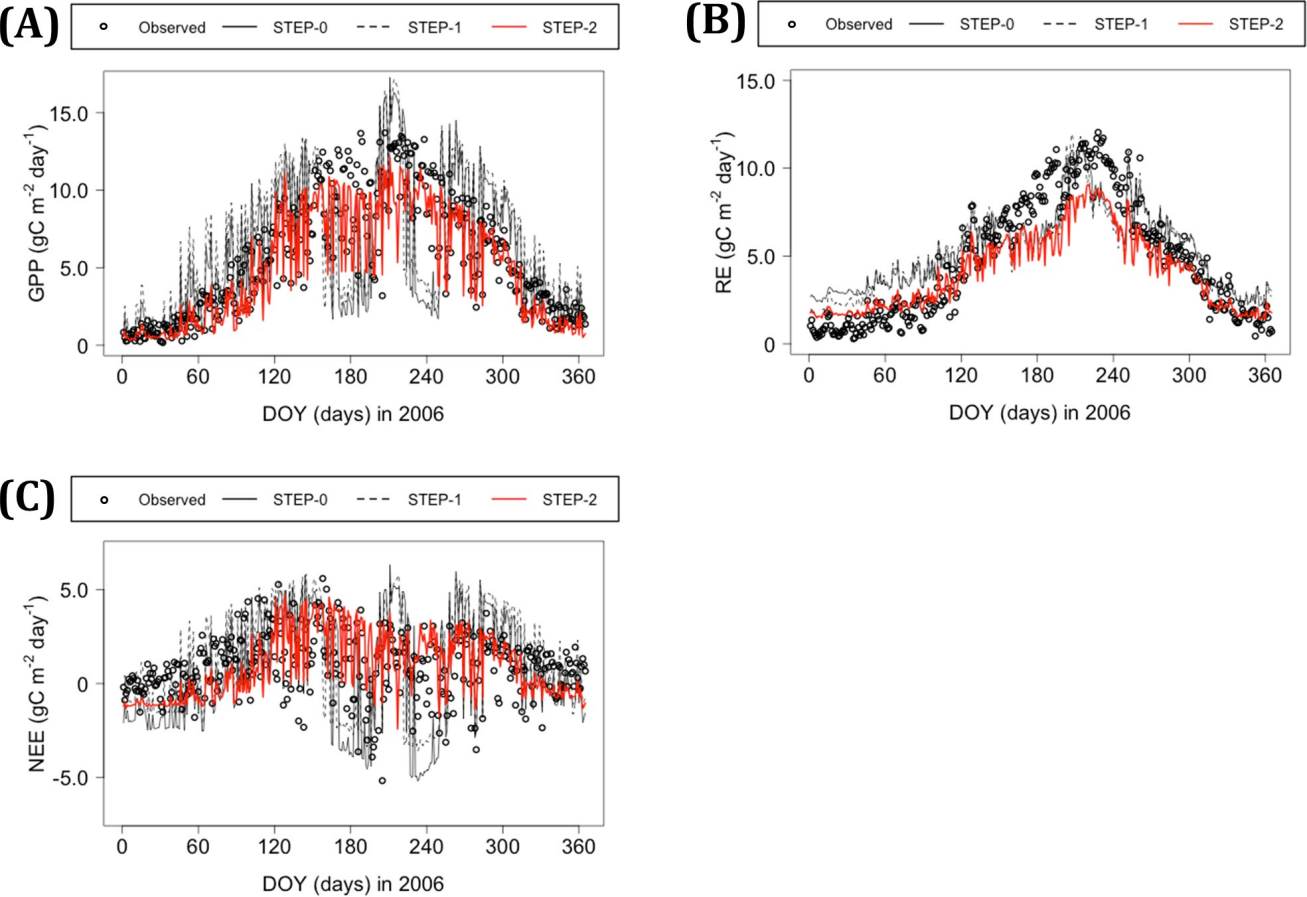
Comparison of simulated flux at the TKC before and after parameterization. (A) Gross primary production (GPP), (B) ecosystem respiration (RE), and (C) net ecosystem exchange (NEE). Gray solid, gray dashed, and red lines are modeled values based on eco-physiological parameters from STEP-0 (parameter set for evergreen needle-leaf forest), and those modified in STEP-1 and STEP-2, respectively.

**Fig 4:**
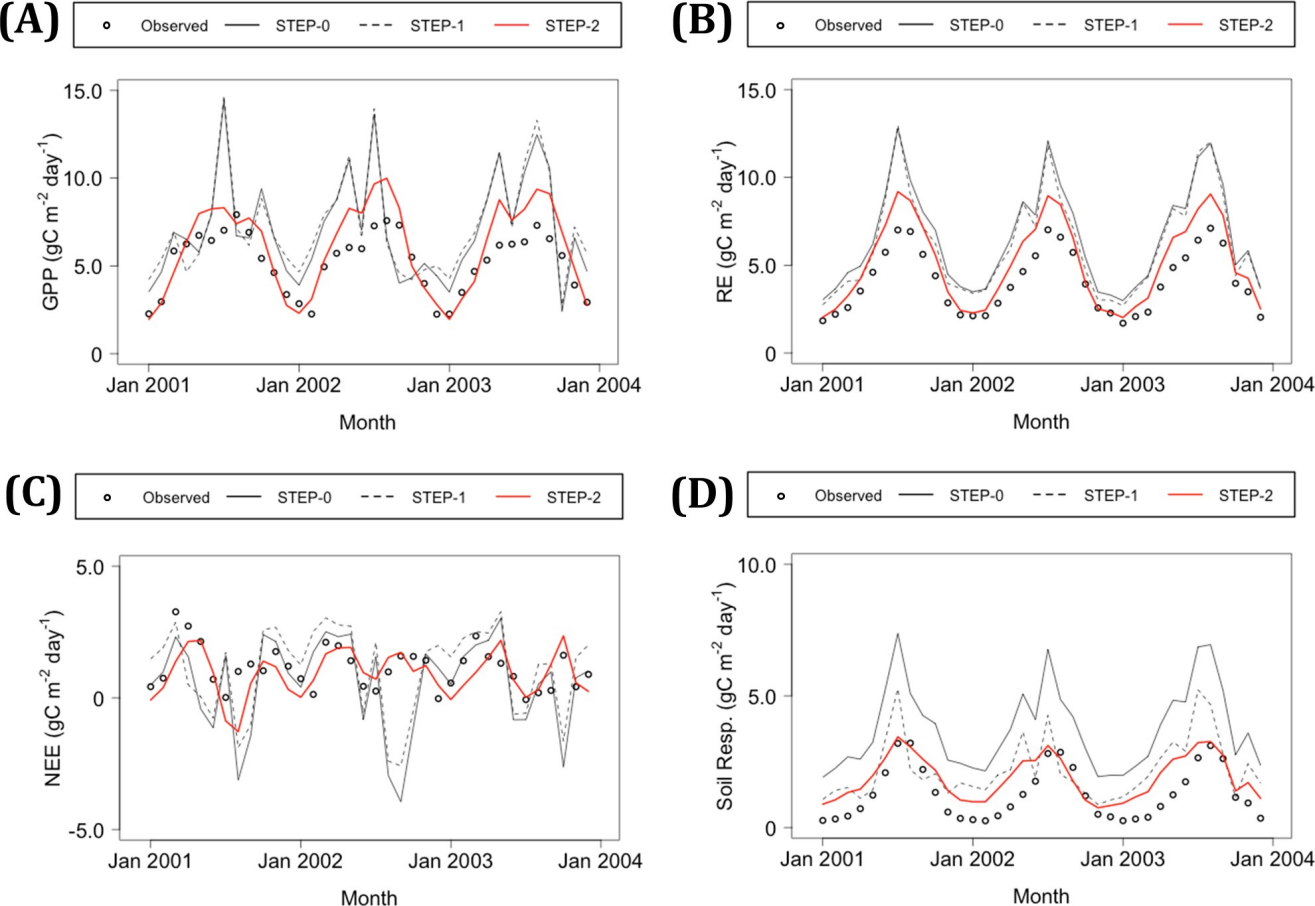
Comparison of simulated flux at the KHW before and after parameterization. (A) Gross primary production (GPP), (B) ecosystem respiration (RE), (C) net ecosystem exchange (NEE), and (D) soil respiration. Gray solid, gray dashed, and red lines are modeled values based on eco-physiological parameters from STEP-0 (parameter set for evergreen needle-leaf forest) and those modified in STEP-1 and STEP-2, respectively.

The modeled fluxes, including GPP and others, improved after two steps of parameterization. The parameters before and after optimization are summarized in Table 1. Three parameters in BO—namely, FLNR, VPD_rsc_start, and VPD_rsc_end—converged (S9 Fig in S1 File). The NRMSE values of modeled GPP were 0.25 and 0.53 in STEP-0 for the TKC and KHW, respectively, which increased slightly in STEP-1 relative to STEP-0 and then decreased in STEP-2 to lower than half the STEP-0 value (Table 4, Figs 3A and 4A). The other model outputs, namely RE, soil respiration, and NEE, which is the only flux with observational data, also improved in STEP-2 (Table 4, Fig 3B and 3C, and Fig 4B–4D).

**Table 4:** Normalized RMSE for modeled flux for the TKC and KHW sites.

	TKC			KHW			
	GPP	RE	NEE	GPP	RE	NEE	Soil resp.
STEP-0	0.25	0.38	0.43	0.53	0.50	0.57	0.87
STEP-1	0.27	0.40	0.41	0.57	0.46	0.52	0.42
STEP-2	0.11	0.34	0.28	0.23	0.22	0.23	0.25

*N* = 365 for the TKC, *N* = 36 for the KHW.

The modeled GPP in the TKC exhibited a sharp decrease followed by an increase in the daily time step during summer in STEP-0, and these variations were removed in STEP-2 (Fig 3A). The modeled RE in the TKC was underestimated in summer, even in STEP-2 (Fig 3B). As for the TKC, the modeled GPP and RE for the KHW exhibited sharp changes with variations in monthly climatic conditions in STEP-0 (Fig 4A and 4B). The effects of parameterization in STEP-1 on GPP and RE in the KHW were small, whereas modeled soil respiration improved. The modeled GPP and RE values became closer to estimates in the KHW in STEP-2, although they were slightly overestimated in summer.

### NPP and ΔNPP

#### Estimated historical trends in NPP

**Fig 5:**
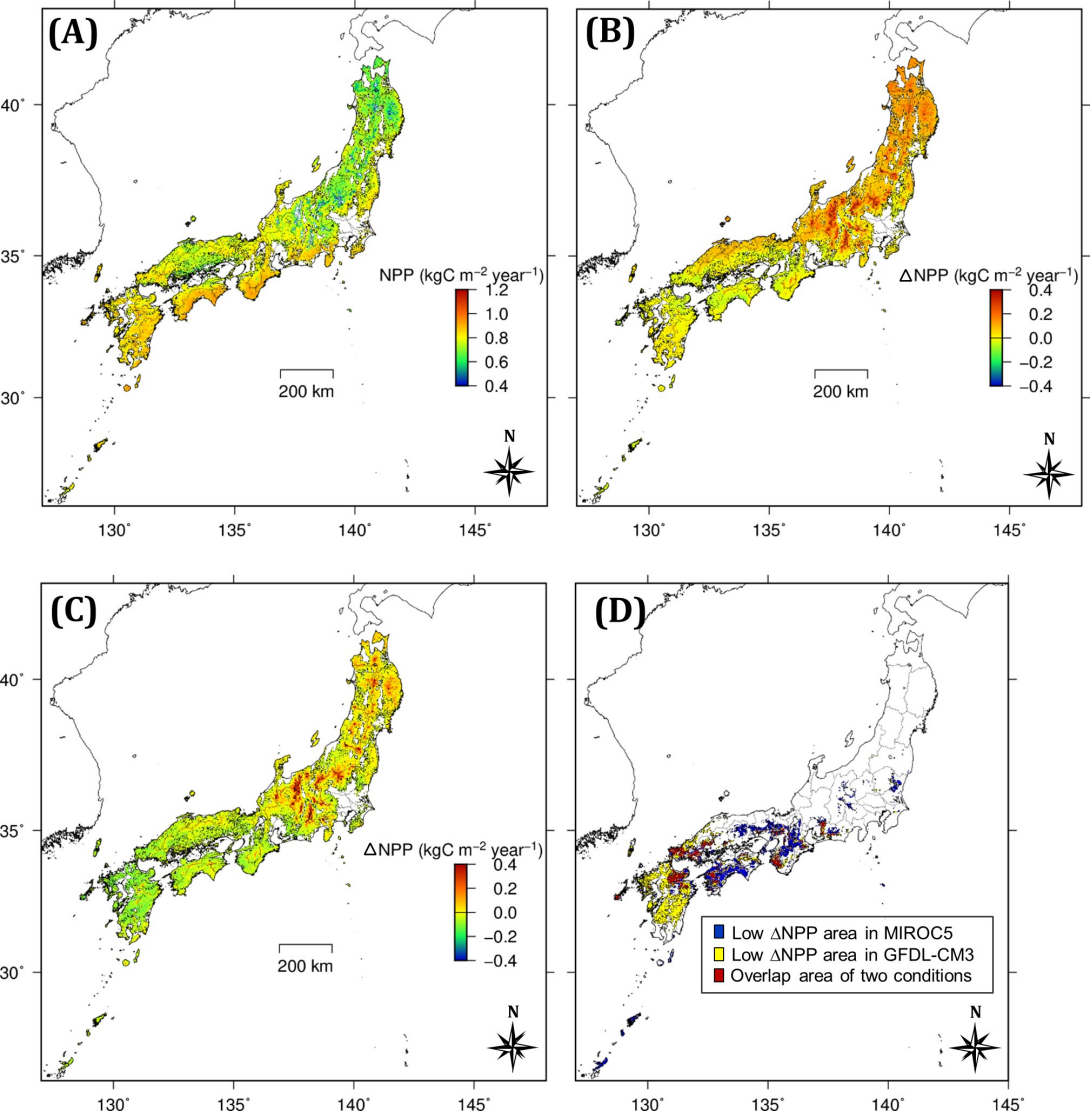
Estimated NPP and ΔNPP of Japanese cedar. (A) NPP for H_2000 from MIROC5, (B) ΔNPP from H_2000 to F_2100 from MIROC5 under RCP2.6, (C) ΔNPP from H_2000 to F_2100 from GFDL-CM3 under RCP2.6, and (D) the NPP-decline area extracted from maps (B) and (C). The blue area of map (D) is based on ΔNPP < −0.02 kgC m^-2^ year^-1^ as indicated in map (B), the yellow area is based on ΔNPP < −0.075 kgC m^-2^ year^-1^ as indicated in map (C), and the red area indicates the overlap area of these two conditions.

**Fig 6:**
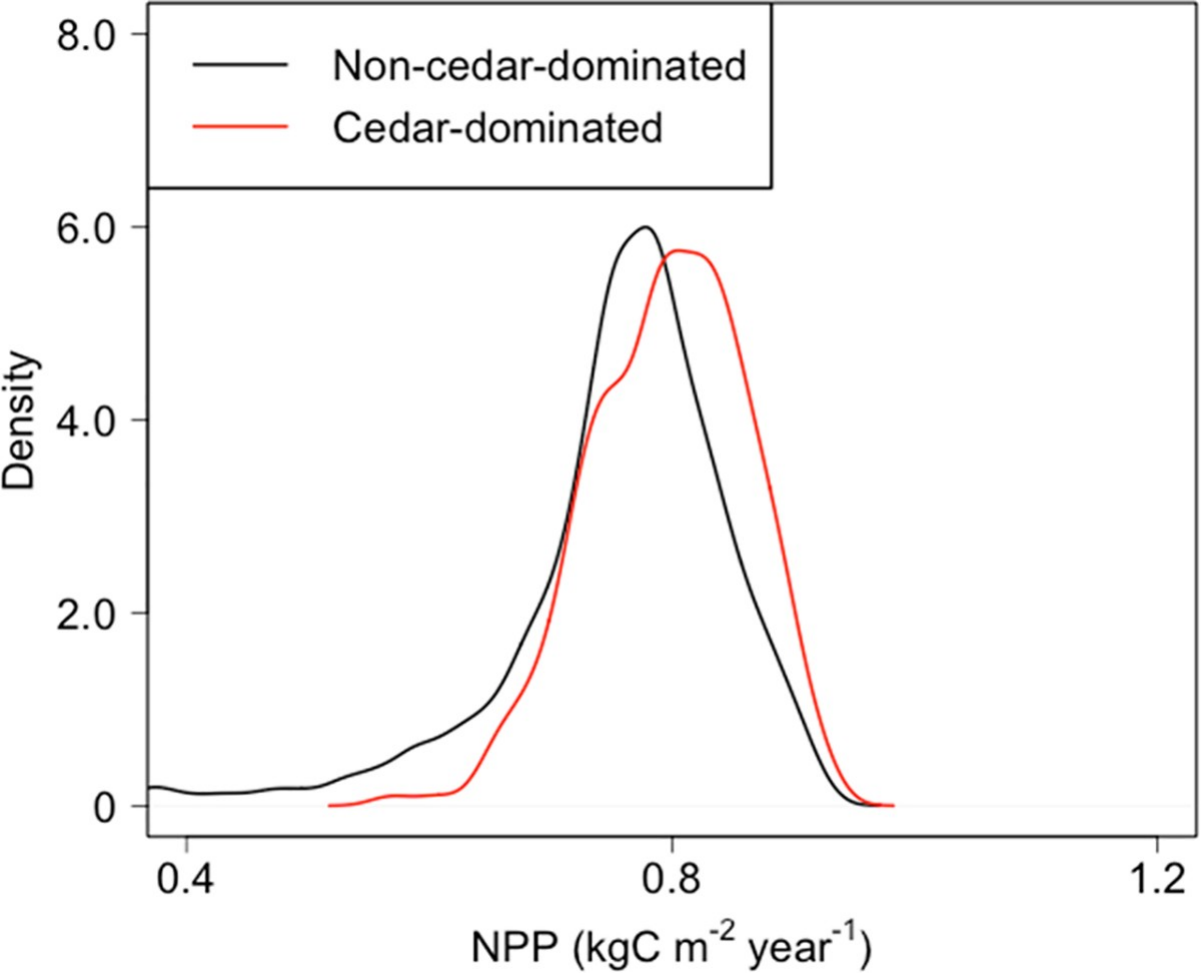
Distribution of modeled NPP in cedar-dominated and non-cedar-dominated areas.

The annual NPP from the five GCMs for H_2000 was 0.77 ± 0.10 kgC m^-2^ year^-1^ (mean ± standard deviation [s.d.]; BBGC_sample) with the highest values in the SW block (Table 5). The annual NPP from MIROC5 for H_2000 was 0.76 ± 0.10 kgC m^-2^ year^-1^, and the trends in regional blocks were quite similar to those generated in the five GCMs (Table 5, Fig 5A). The range of annual NPP from MIROC5 at STEP-2 was significantly higher in currently cedar-dominated mesh points (0.80 ± 0.06 kgC m^-2^ year^-1^, 26% of total area) than in non-cedar-dominated (0.75 ± 0.11 kgC m^-2^ year^-1^, 74% of total area) forests (p <0.001; Fig 6).

**Table 5:** NPP, ΔNPP, and proportion of NPP-decline area.

		NPP in H_2000			ΔNPP in F_2100						NPP-decline area	
GCM	Block				RCP2.6			RCP8.5			RCP2.6	RCP8.5
		(kgC m^-2^ year^-1^)									(%)	
5GCMs	SW	0.81	±	0.06	0.01	±	0.05	0.10	±	0.08	37	16
	CT	0.77	±	0.12	0.05	±	0.08	0.15	±	0.13	23	8
	NE	0.72	±	0.09	0.07	±	0.06	0.17	±	0.09	10	2
	Total	0.77	±	0.10	0.04	±	0.07	0.14	±	0.11	24	9
MIROC5	SW	0.81	±	0.06	0.01	±	0.04	0.13	±	0.04	49	0
	CT	0.76	±	0.12	0.06	±	0.09	0.17	±	0.12	25	0
	NE	0.71	±	0.09	0.10	±	0.06	0.19	±	0.09	4	0
	Total	0.76	±	0.10	0.06	±	0.08	0.16	±	0.09	27	0

Mean ± s.d.; SW, CT, and NE indicate the southwestern, central, and northeastern blocks, respectively.

The seasonal NPP estimates from the five GCMs for H_2000, in daily terms, were 3.21± 0.47 and 0.98 ± 0.50 gC m^-2^ day^-1^ during the warm and cool seasons, respectively, and the mean values in three regional blocks, SW, CT, and NE, were 3.05, 3.24, and 3.35 gC m^-2^ day^-1^ (mean from five GCMs) for the warm season and 1.40, 0.93, and 0.59 gC m^-2^ day^-1^ for the cool season, respectively. These seasonal trends in NPP from MIROC5 were similar to those obtained from the five GCMs.

#### Estimated ΔNPP under the RCP2.6 and RCP8.5 scenarios

**Fig 7:**
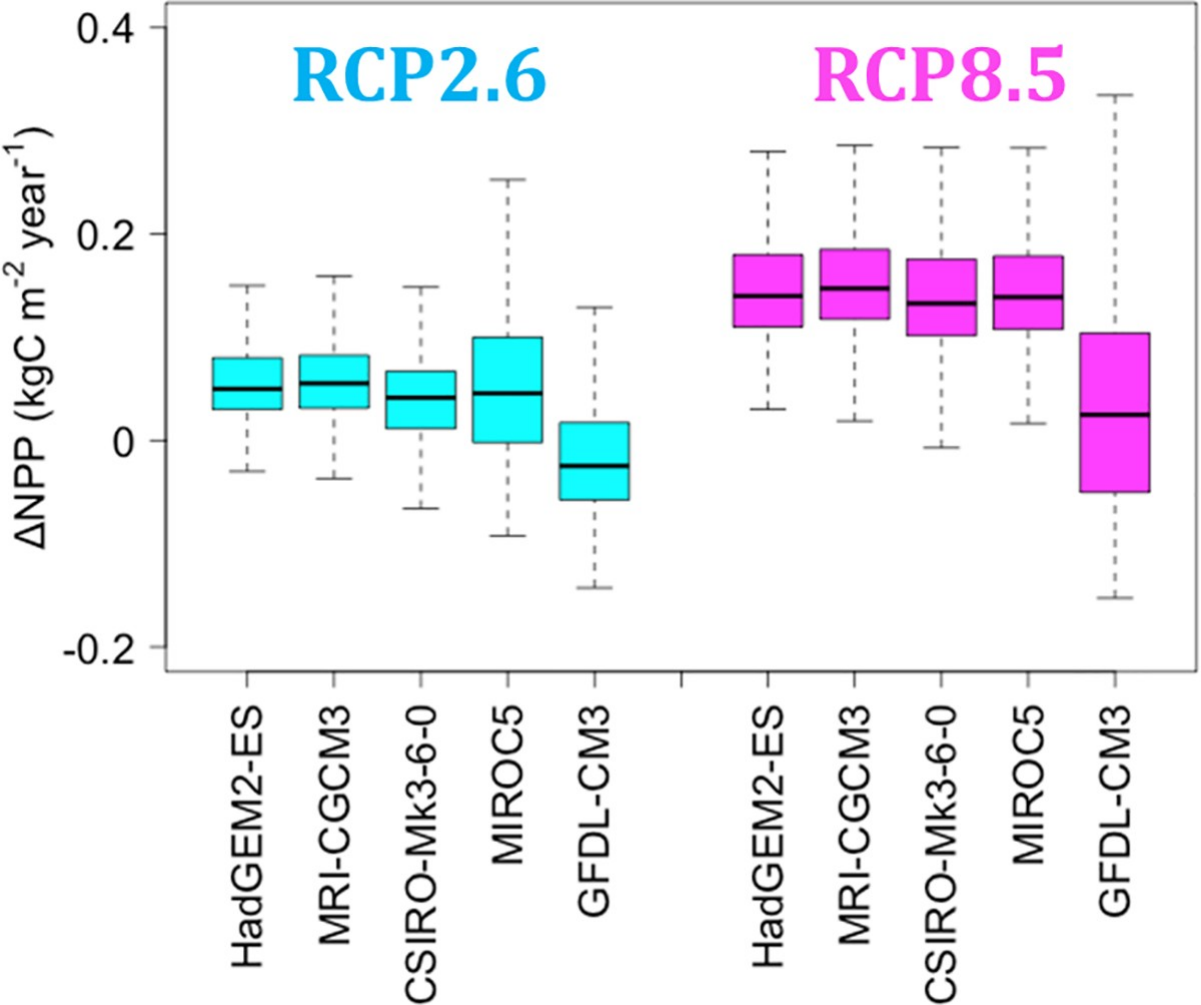
Trends in ΔNPP according to five GCMs. Comparison between H_2000 and F_2100. The outliers of ΔNPP are not shown in the figure, as the resulting wide ranges obscure the differences among GCMs.

The changes in NPP (ΔNPP) according to the five GCMs were 0.04 ± 0.07 and 0.14 ± 0.11 kgC m^-2^ year^-1^ (mean ± s.d.; BBGC_sample) under the RCP2.6 and RCP8.5 scenarios, respectively, and the ΔNPPs were lower in the SW region than in the CT and NE regions (Table 5). Decreases in NPP (negative ΔNPP) in RCP2.6 were predicted more frequently with MIROC5 (27% of total area) and GFDL-CM3 (67%, Fig 7), and the NPP-decline area was distributed primarily in the western part of Japan (Fig 5B–5D). Negative ΔNPP under RCP8.5 was predicted solely with GFDL-CM3 (Fig 7).

The mean ΔNPP from the five GCMs under RCP2.6 was negative and positive in the warm and cool seasons, respectively, and was especially low in the warm season in the SW block (Table 6). The mean ΔNPP from the five GCMs under RCP8.5 was much higher than that under RCP2.6 during the cool season.

**Table 6:** Seasonality of ΔNPP in F_2100.

		ΔNPP in RCP2.6							ΔNPP in RCP8.5					
GCM	Block	Warm			Cool				Warm			Cool		
		(gC m^-2^ day^-1^)												
5GCMs	SW	-0.18	±	0.32	0.23	±	0.14		-0.02	±	0.52	0.56	±	0.25
	CT	0.02	±	0.49	0.28	±	0.14		-0.10	±	0.82	0.91	±	0.23
	NE	0.14	±	0.39	0.26	±	0.12		0.00	±	0.60	0.91	±	0.18
	Total	-0.01	±	0.43	0.26	±	0.13		-0.04	±	0.66	0.79	±	0.27

Mean ± s.d.; SW, CT, and NE indicate the southwestern, central, and northeastern blocks, respectively. Warm and Cool indicate the warm (May to October) and cool (November to April) seasons, respectively.

### Recorded and modeled NPP and biomass C relative to stand age

**Fig 8:**
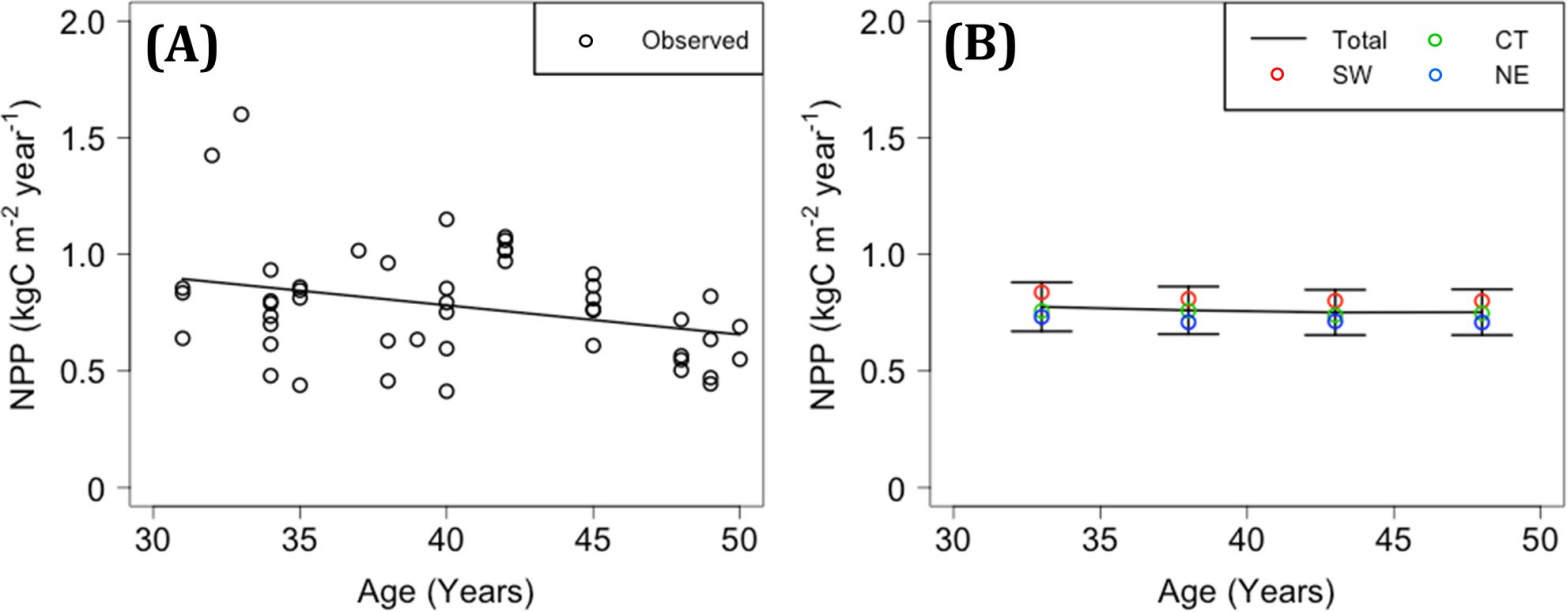
Changes in observed and modeled NPP with stand age. (A) Observed NPP. Open circles indicate records in the PTDB, and the solid line represents the regression with stand age. (B) Modeled NPP. The solid lines show the mean and standard deviation in the total area for every 5-year bin from 31 to 50 years. SW, CT, and NE indicate mean values of the southwestern, central, and northeastern blocks, respectively.

A total of 48 records of NPP for 31–50 year-old stands, aged 40.3 years on average, were extracted from among 116 records for 5–70 year-old stands in the PTDB. The NPP for 31–50-year-old stands was 0.78 ± 0.24 kgC m^-2^ year^-1^ and exhibited a decreasing trend with stand age (*p* <0.05; Fig 8A). Meanwhile, the mean value of modeled NPP in STEP-2, based on the historical trend estimated in MIROC5, ranged from 0.75 to 0.78 kgC m^-2^ year^-1^ for every 5-year bin from 31 to 50 years of age, and its change with stand age was unclear (Fig 8B).

**Fig 9:**
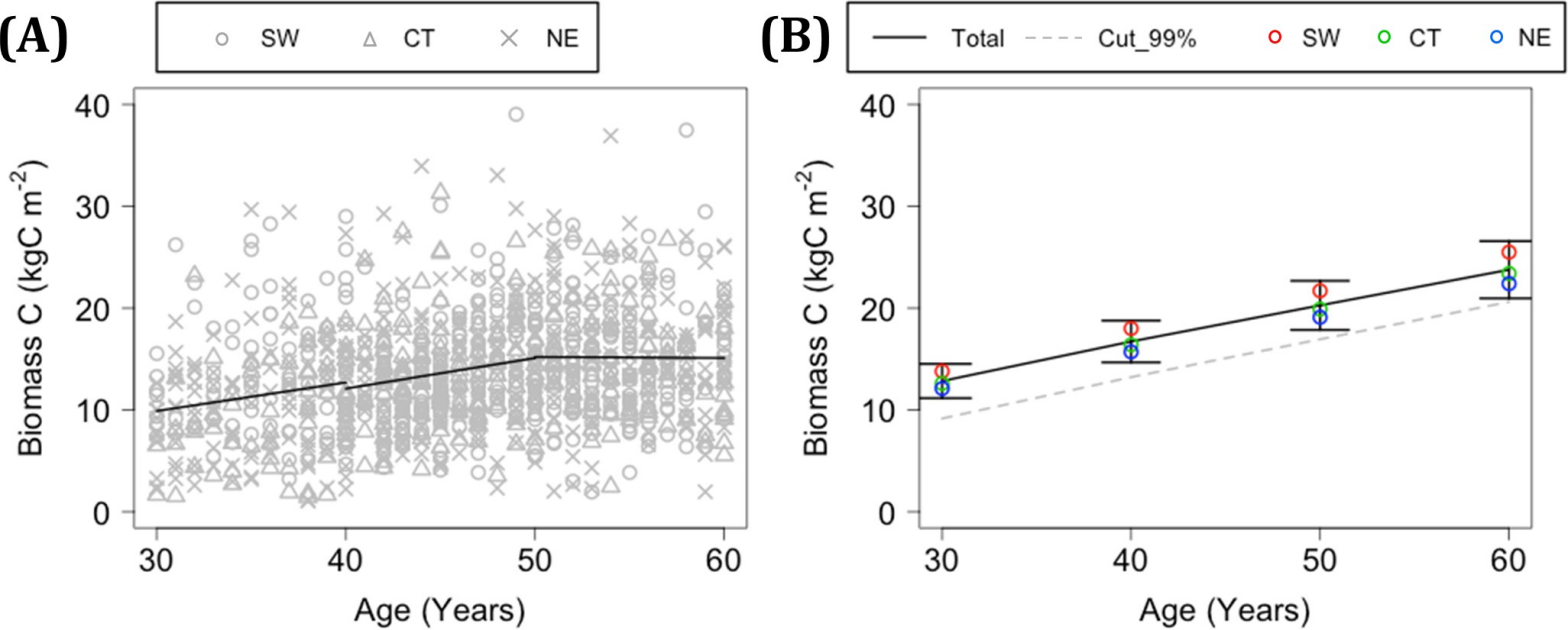
Changes in estimated and modeled biomass C with stand age. (A) Estimated biomass C based on wood volume in the NFI. The solid line represents the regression of biomass C with age for 10-year bins. (B) Modeled biomass C. The black solid line indicates the mean and standard deviation of biomass C in the total area at 10-year intervals. The gray dotted line indicates mean biomass C based on a cutting ratio of 99% in existing forests. SW, CT, and NE indicate mean values of the southwestern, central, and northeastern blocks, respectively.

In total, 1094 records of wood volume for 30–60-year-old stands were extracted from the NFI. The temporal trends in stand biomass C, which was converted from wood volume based on a BEF, were determined for every 10-year period as the coefficient of a single regression with stand age; these values were 0.279, 0.303, and −0.01 kgC m^-2^ year^-1^ for stand ages of 30–40, 40–50, and 50–60 years, respectively (Fig 9A). The differences in stand biomass C among regional blocks were unclear in this dataset, except that higher biomass was observed in the SW region relative to the CT and NE regions at age 30–40 years (*p* <0.001). Meanwhile, the mean increments of modeled stand biomass C were 0.388, 0.355, and 0.350 kgC m^-2^ year^-1^ for stand ages of 30–40, 40–50, and 50–60 years, respectively, indicating a relatively monotonic increase in stand biomass (Fig 9B). The modeled stand biomass C was generally higher in the SW block compared to the CT and NE blocks.

## Discussion

### Validity and limitations of estimating NPP through two-step parameterization

The parameterization conducted in this study improved the estimation of the physiological responses of Japanese cedar plantations growing under humid climatic conditions in East Asia. We confirmed that changes in eco-physiological parameters in STEP-1, which were mainly based on PTDB data, including the C:N ratio of leaves, SLA, and turnover of fine roots, rarely contributed to improving the simulated seasonality of GPP in daily or monthly outputs for the TKC and KHW. Meanwhile, changes in FLNR and the VPD-stomatal relationship due to BO in STEP-2 sharply improved simulated GPP at those sites. As a result, our estimation of annual NPP in the TKC in 2006 approached 0.79 kgC m^-2^ year^-1^, which is the biometric estimate over 4 years (2005–2009) as reported by Yashiro et al. [61], rising from 0.65 kgC m^-2^ year^-1^ in STEP-0 to 0.73 kgC m^-2^ year^-1^ in STEP-2. The VPD-stomatal relationship may be the key factor for simulating seasonality rather than FLNR, which instead altered the overall range of NPP [40]. The range of the VPD-stomatal relationship in STEP-2 was 135–1824 Pa, which included the entire ranges of mean daytime-VPD in the warm seasons of 302–1257 Pa for H_2000 and 428–1497 Pa for F_2100 from MIROC5 under RCP8.5. This range is quite low compared to that presented in BBGC for Japanese cypress (610–4100 Pa; [9]), which exhibits more water-saving characteristics than Japanese cedar when the soil water supply is limited [62], making the modeled stomatal behavior of Japanese cedar more sensitive to increases in VPD within the climatic range of Japan. The observed range of the VPD-stomatal relationship at the canopy level ([42, 63–65] supports adjustment to a substantially lower range of VPD compared to that in STEP-0 (930–4100 Pa). We optimized these parameters by simultaneously improving modeled GPP at two sites under different climatic conditions and timescales, the TKC and KHW, and showed that the weighting of the likelihood function in BO was effective.

The modeled NPP results for H_2000 were considered valid from the perspective of their geographical distribution. The mean NPP for H_2000 for stands aged 36–40 years was similar to that for stands aged 31–50 years in the PTDB (Fig 8A and 8B). The NPP and stand biomass C for 36–40-year-old stands for H_2000 were higher in the SW and lower in the NE region (Figs 5A, 8B and 9B). This result was consistent with statistical trends showing that the growth rate in terms of height of Japanese cedar is high in the southern and Pacific Ocean coastal regions of Japan, especially during the initial stage of growth [18]. Ito [10] estimated NPP in various biomes in East Asia using a C cycle model and obtained high values for evergreen needle-leaf forests in the southern and coastal regions of Japan. Hashimoto et al. [66] inversely estimated NPP in forested areas of Japan, not only for Japanese cedar forests, using a soil C stock dataset and the RothC model, and obtained higher values in southwestern regions compared to northeastern regions. Mitsuda [5] estimated NPP in Japanese cedar plantations on Kyushu Island (covering approximately 40% of the SW block) using the 3-PG model and obtained high values in the southern area, in accordance with this study. Regardless of the spatial validity of estimated NPP, it is important to note that we might not have sufficiently simulated the age dependency of NPP. Although the observed NPP values in the PTDB are not successional data for a single stand, they exhibited a decreasing trend with stand age from 31 to 50 years (Fig 8A), whereas the average NPP values for H_2000 and F_2100 at 5-year intervals exhibited a relatively monotonous trend over that period (Fig 8B). The estimated biomass C from NFI exhibited an increasing trend with stand age for at least up to 50 years, which probably includes the negative effect of harvesting at higher stand ages (Fig 9A). Meanwhile, the modeled biomass C in this study increased monotonically from 30 to 60 years of stand age (Fig 9B). In summary, we could not validate the modeled high growth rate of old Japanese cedar in stands >50 years of age, and it would be inappropriate to apply the model parameterized in this study to stands much older or younger than 40 years. The age dependency of NPP is a sensitive factor that complicates the modeling and assessment of climate impacts on planted forests. In this study, the weak dependency of NPP on stand age enabled comparison of NPP at a fixed age, namely 36–40 years, between current and future climatic conditions.

### Difference in estimates of ΔNPP between the RCP2.6 and RCP8.5 scenarios

**Fig 10:**
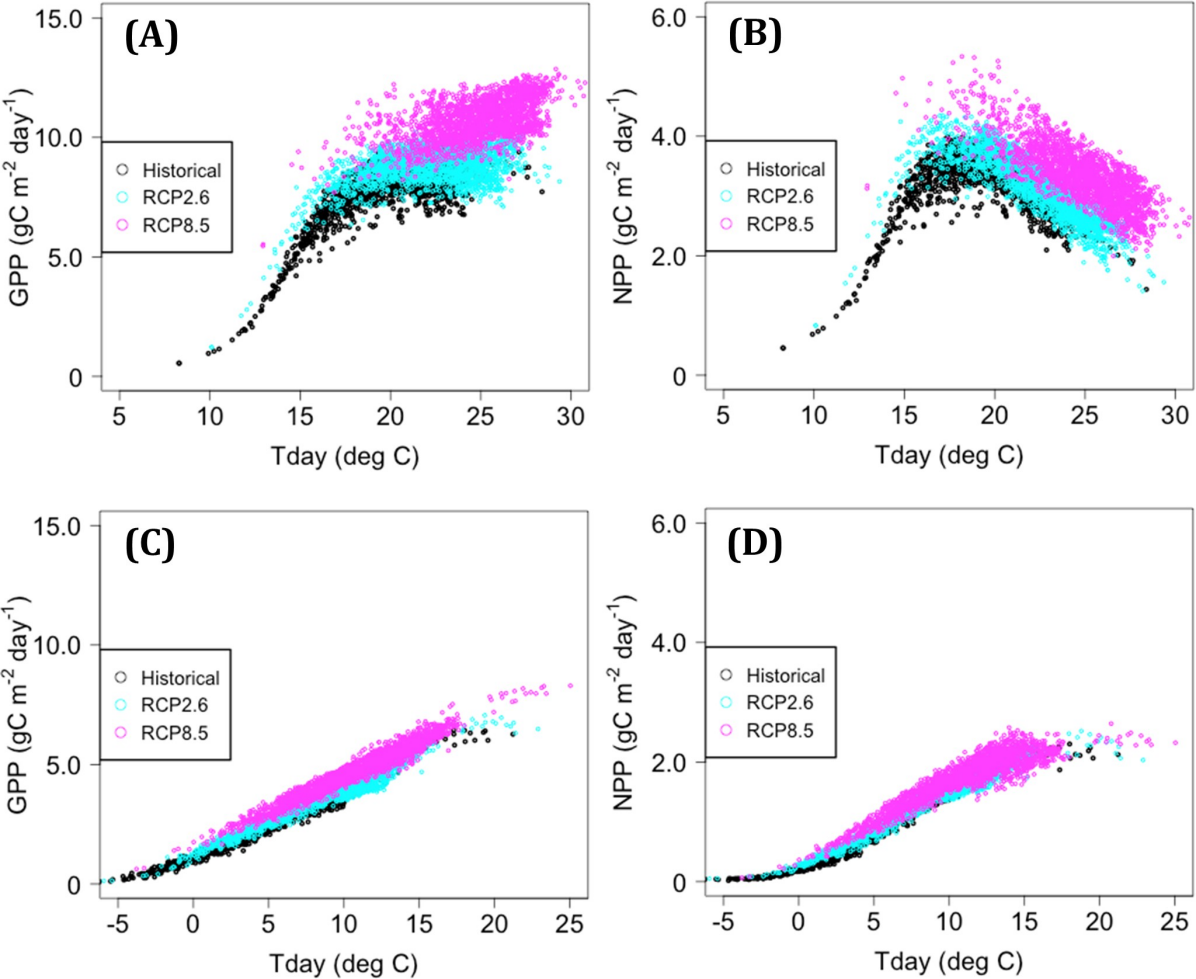
Relationships of GPP and NPP with daytime-mean air temperature. Tday, daytime-mean air temperature. (A) GPP in the warm season, (B) NPP in the warm season, (C) GPP in the cool season, and (D) NPP in the cool season.

The mean ΔNPP for F_2100 was generally lower under the RCP2.6 scenario than under RCP8.5. This difference was likely due to mitigation of the decrease in modeled GPP during warm seasons related to the high CO_2_ concentration in RCP8.5. The modeled relationship between GPP and Tday (mean air temperature in daytime) during the cool season and that between NPP and Tday exhibited similar trends of temperature dependency for both H_2000 and F_2100 (based on MIROC5; Fig 10C and 10D). On the other hand, the GPP-Tday and NPP-Tday relationships during the warm season shifted upward from the historical climate to RCP2.6 and further to RCP8.5 (Fig 10A and 10B). In an additional test of the RCP8.5 climate scenario with the CO_2_ concentration reduced to the level of RCP2.6, the modeled GPP and NPP for F_2100 were sharply reduced, and the percentage of the total area with negative ΔNPP increased from 0% under the original RCP8.5 conditions to 45%. Similar results have been reported in previous studies of coniferous forests in East Asia, mainly in China. When the effect of elevated CO_2_ was considered, increased NPP was predicted more frequently under RCP8.5 than under RCP2.6 for tree species such as *Pinus tabulaeformis* [67] and *Abies fabri* [68], although if that effect was neglected, the risk of decreased productivity became high under RCP8.5, as noted for *Larix olgensis* [69] and *A*. *fabri* [68]. A positive effect of elevated CO_2_ on GPP could be detected at the global scale, even under the current climatic conditions [70]. Down-regulation of photosynthesis under elevated CO_2_ conditions, leading to an observed rate lower than that estimated by Farquhar’s model, has not been confirmed for mature stands of Japanese cedar. However, Hiraoka et al. [71] reported that the photosynthetic rate of 1-year-old cuttings of Japanese cedar increased under elevated CO_2_ conditions, whereas the maximum carboxylation rate decreased. Thus, the increase in NPP under highly elevated CO_2_ conditions may be lower than that predicted in this study. To account for this down-regulation in future modeling of photosynthesis, reduction of Max_sc through scaling of CO_2_ concentration ([72, 73]) may be an effective strategy, although the sensitivity analysis in this study indicated that reducing Max_sc did not necessarily decrease modeled NPP under the RCP8.5 scenarios (S8 Text in S1 File).

### Geographic characteristics of the modeled decrease in NPP in Japanese cedar plantations

**Fig 11:**
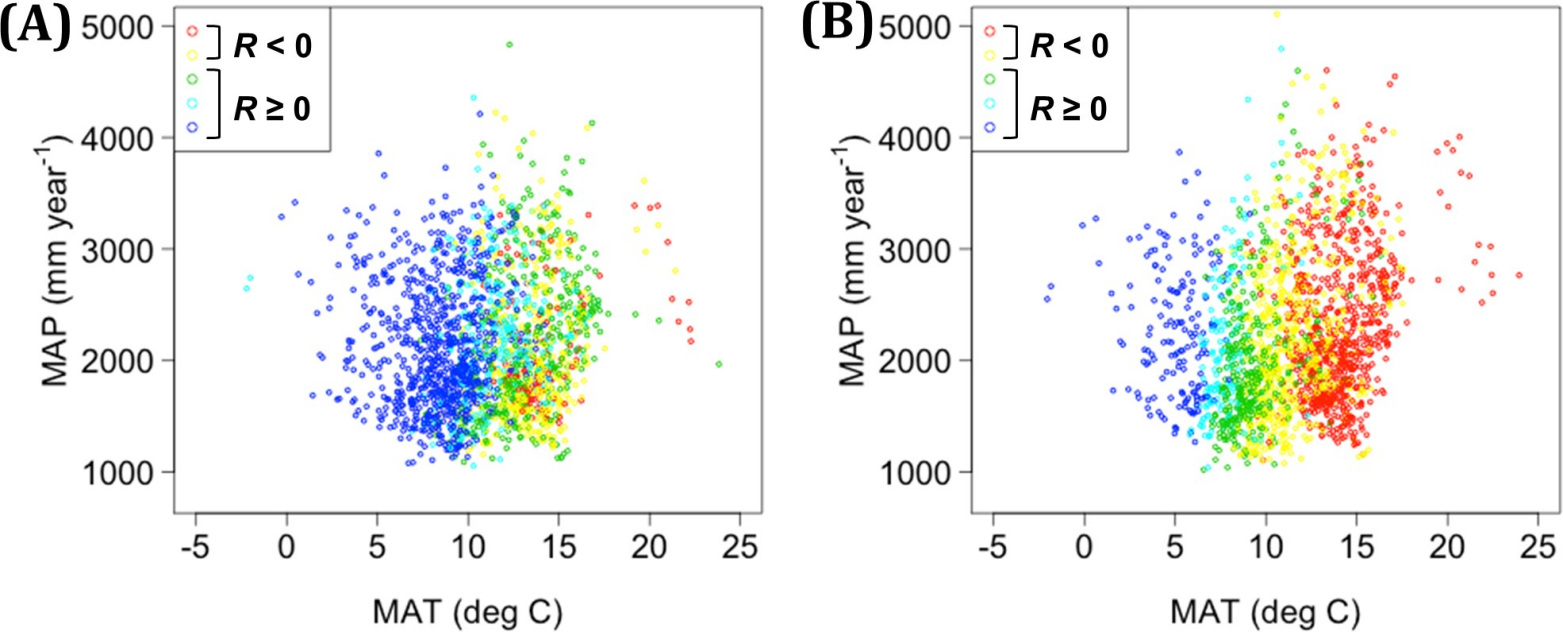
Distribution of the ΔNPP-to-ΔMAT ratio in relation to MAT and MAP under the current climate. Results from (A) MIROC5 and (B) GFDL-CM3, based on the RCP2.6 scenario. *R* represents the ΔNPP-to-ΔMAT ratio. Red (*R* < −0.015) and yellow (−0.015 ≤ *R* <0) indicate decreases in NPP with increased temperature, whereas green (0 ≤ *R* < 0.015), light blue (0.015 ≤ *R* < 0.03), and blue (*R* ≥ 0.03) indicate increases in NPP.

Here, we focus on the area of decreased NPP (NPP-decline area) under the RCP2.6 scenario, considering the potential risk of decreased productivity of Japanese cedar plantations. The NPP-decline area in this study was concentrated in the SW region (Fig 5D), in accordance with previous studies reporting high vulnerability of Japanese cedar in western Japan to a slowly warming climate [5, 22]. The geographic distribution of negative annual ΔNPP was derived from the decrease of NPP in the warm season, rather than its increase in the cool season (Table 6). The area that is sensitive to increasing temperature, evaluated based on the ratio of ΔNPP to ΔMAT, was characterized mainly by MAT in the historical dataset. Negative values of ΔNPP/ΔMAT were predicted more frequently by two GCMs, MIROC5 and GFDL-CM3, in this study, and were dominant when MAT for H_2000 exceeded approximately 13 deg C (Fig 11), whereas their relationship with MAP was unclear. Risk assessment of the NPP-decline area at the continental scale showed that the risk tended to be high in the southern area, where the level of NPP in the current climate is high [74–76].

In summary, we first evaluated the vulnerability of Japanese cedar plantations to a changing climate by modeling stand productivity at the national scale in Japan. Despite geographic variation in ΔNPP, the mean values of ΔNPP under RCP2.6 and RCP8.5 were positive for all but one GCM, and the overall vulnerability to decreasing NPP was predicted to be relatively low. These results may help with recommending the best management options for planted forests in other regions with warm and humid climates. However, the consequences of decreasing NPP in the warm season were not fully examined in this study, such as the possible linkage between reduced non-structural C and mortality [28]. Monitoring of planted forests should be enhanced in southwestern Japan. The part of this region with high MAT under the current climate may require the development of new varieties of Japanese cedar or a shift toward other tree species such as Japanese cypress to properly address the vulnerability of planted forests to warming summers in the future.

## Supporting information

S1 File (PDF)

S2 File (ZIP)
