# Supplementary material for: Estimating spatial variation in the effects of climate change on the net primary production of Japanese cedar plantations based on modeled carbon dynamics
Source: PLoS One. 2021 Feb 17;16(2):e0247165. doi: 10.1371/journal.pone.0247165 (PMC7888599; doi:10.1371/journal.pone.0247165)
Supplement: S1 File — (PDF) [file pone.0247165.s001.pdf]

Title

Estimating spatial variation in the effects of climate change on the net primary production of Japanese cedar plantations based on modeled carbon dynamics

Authors

Jumpei Toriyama[1,*], Shoji Hashimoto[2,3], Yoko Osone[2], Naoyuki Yamashita[2], Tatsuya Tsurita[2], Takanori Shimizu[4], Taku M. Saitoh[5], Shinji Sawano[6], Aleksi Lehtonen[7], Shigehiro Ishizuka[2]

Affiliation

[1] Kyushu Research Center, Forestry and Forest Products Research Institute (FFPRI), Kumamoto-city, Kumamoto, Japan

[2] Department of Forest Soils, FFPRI, Tsukuba, Ibaraki, Japan

[3] Graduate School of Agricultural and Life Sciences, The University of Tokyo, Bunkyo-ku, Tokyo, Japan

[4] Department of Disaster Prevention, Meteorology and Hydrology, FFPRI, Tsukuba, Ibaraki, Japan

[5] River Basin Research Center, Gifu University, Gifu-city, Gifu, Japan

[6] Hokkaido Research Center, FFPRI, Sapporo, Hokkaido, Japan

[7] Natural Resources Institute Finland, Helsinki, Finland

* Corresponding author (JT)

E-mail: jtori@affrc.go.jp

DOI

10.1371/journal.pone.0247165

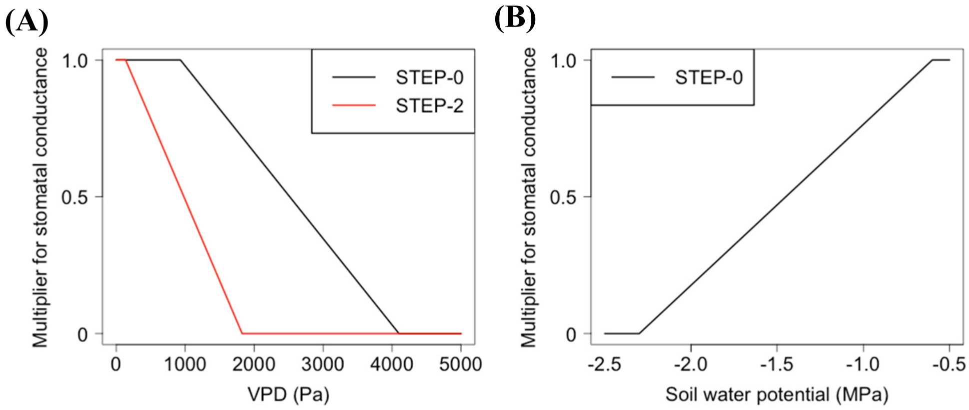

S1 Fig. Multipliers of stomatal conductance

(A) Vapor pressure deficit (VPD) and (B) soil water potential.

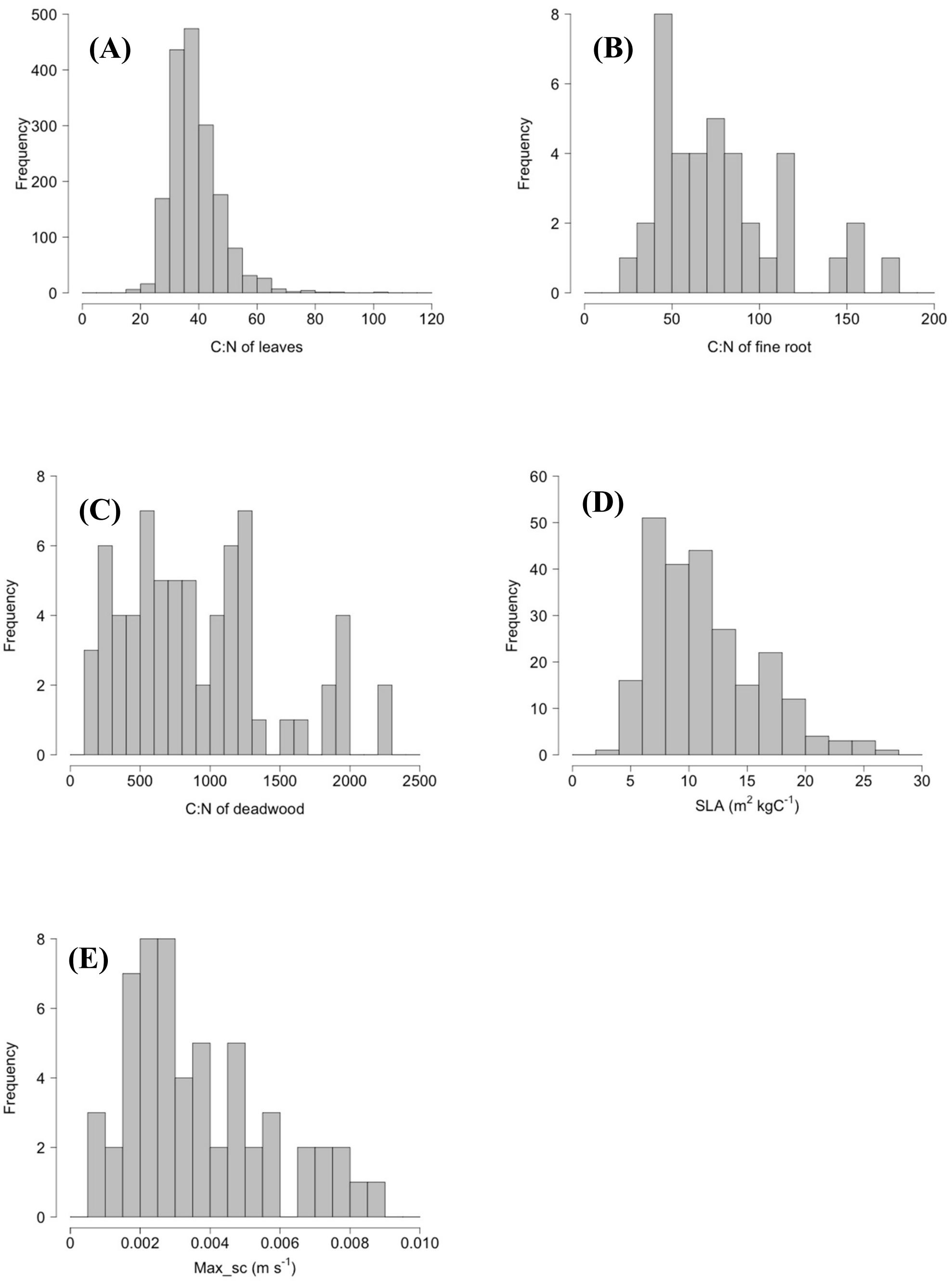

S2 Fig. Histogram of observed data for five eco-physiological parameters in the plant trait database

(A) C:N ratio of leaves, (B) C:N ratio of fine roots, (C) C:N ratio of deadwood, (D) specific leaf area (SLA), and (E) maximum stomatal conductance (Max_sc).

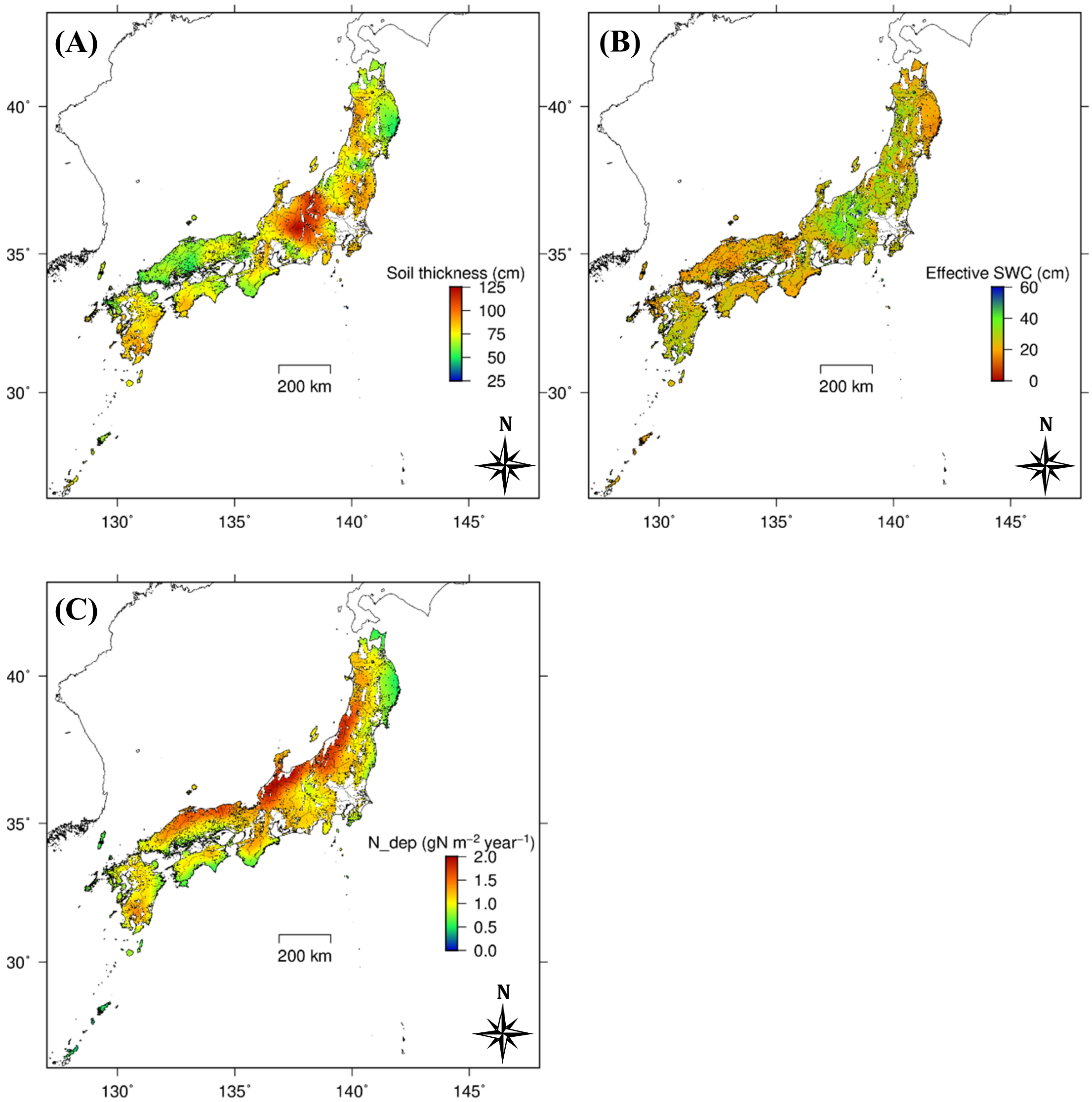

S3 Fig. Map of spatial distribution of site parameters

(A) Soil thickness, (B) the effective soil water-holding capacity of the soil profile, and (C) nitrogen deposition rate. The effective soil water-holding capacity of the soil profile is the product of soil thickness and the difference between the van Genuchten (vG) parameters θ_s and θ_r.

The data is deposited to a public repository (doi: 10.5281/zenodo.4505671).

S4 Table. The vG parameters applied in this study

No	Soil code	A horizon				B horizon			
		θs (θ^3 θ^{-3})	*θr*	*α* (MPa^{-1})	*n*	*θs* (θ^3 θ^{-3})	*θr*	*α* (MPa^{-1})	*n*
1	PZ_dry	0.56	0.10	3173	1.20	0.55	0.23	2239	1.27
2	PZ_wet	0.56	0.10	3173	1.20	0.55	0.23	2239	1.27
3	BFS_dry	0.55	0.27	1126	1.41	0.56	0.29	878	1.44
4	BFS_moist	0.67	0.29	1587	1.32	0.66	0.37	983	1.43
5	BFS_wet	0.58	0.33	1209	1.49	0.58	0.33	1252	1.41
6	dBFS	0.67	0.29	1587	1.32	0.66	0.37	983	1.43
7	ryBFS	0.67	0.29	1587	1.32	0.66	0.37	983	1.43
8	RY	0.64	0.19	7134	1.20	0.47	0.25	2756	1.21
9	BL	0.71	0.38	659	1.48	0.70	0.40	568	1.52
10	lBL	0.71	0.38	659	1.48	0.70	0.40	568	1.52
11	DR	0.64	0.19	7134	1.20	0.47	0.25	2756	1.21
12	PT	0.79	0.00	398	1.25	0.79	0.00	398	1.25
13	IM	0.50	0.00	7382	1.12	0.50	0.00	2424	1.12

S5 Table. Changes in VPD and solar radiation

GCM	Block	VPD in H_2000	∆VPD in F_2100 RCP2.6	∆VPD in F_2100 RCP8.5	Srad in H_2000	∆Srad in F_2100 RCP2.6	∆Srad in F_2100 RCP8.5
		(Pa)			(W m^{-2})		
5GCMs	SW	673 ± 86	68 ± 29	203 ± 67	312 ± 16	-7 ± 4	-14 ± 5
	CT	606 ± 109	70 ± 30	205 ± 66	321 ± 28	-9 ± 7	-21 ± 11
	NE	524 ± 87	62 ± 27	191 ± 59	307 ± 22	-11 ± 6	-25 ± 10
	Total	602 ± 112	67 ± 29	200 ± 65	314 ± 23	-9 ± 6	-20 ± 10
MIROC5	SW	666 ± 84	89 ± 16	163 ± 25	317 ± 16	-10 ± 3	-17 ± 4
	CT	597 ± 105	95 ± 15	192 ± 30	326 ± 28	-10 ± 7	-22 ± 11
	NE	521 ± 84	71 ± 22	175 ± 25	313 ± 22	-14 ± 6	-27 ± 10
	Total	595 ± 109	85 ± 21	177 ± 29	319 ± 23	-11 ± 6	-22 ± 10

Mean ± s.d.; VPD and solar radiation (Srad) were estimated on a daytime basis. SW, CT, and NE indicate the southwestern, central, and northeastern blocks, respectively.

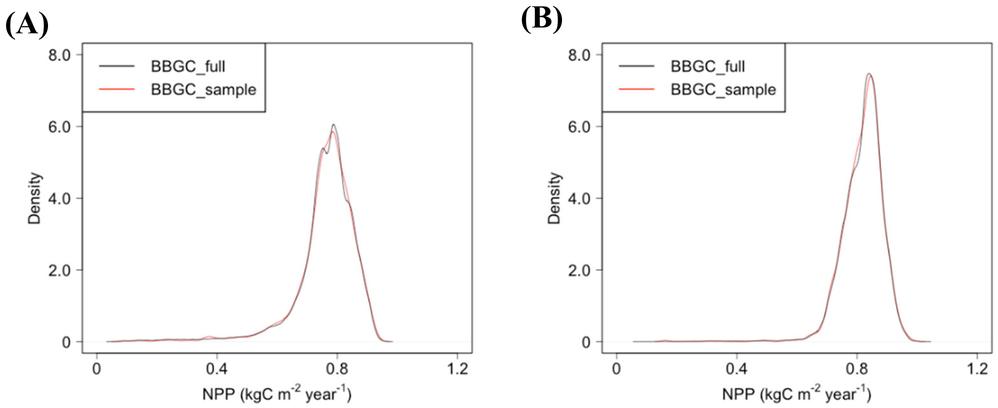

S6 Fig. Distribution of calculated net primary production (NPP) in the BBGC_full and BBGC_sample datasets

(A) Calculated NPP in the 1996–2000 period (H_2000) and (B) predicted NPP for 2096–2100 (F_2100) under RCP2.6 according to MIROC5.

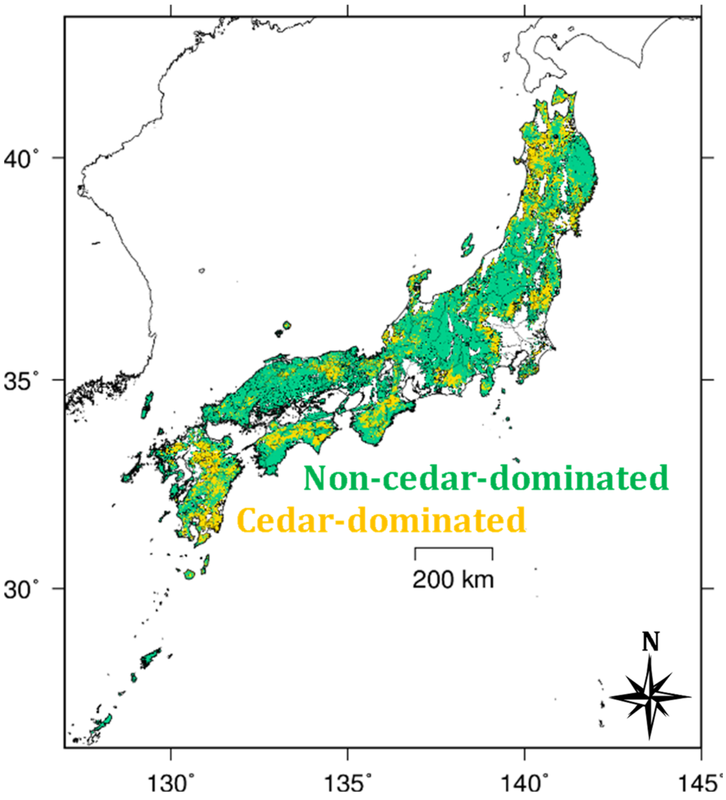

S7 Fig. Map of cedar-dominated and non-cedar-dominated areas

S8 Text. Sensitivity of NPP to eco-physiological and site parameters

Methodology

In this study, the sensitivity of mean NPP of the total area to six eco-physiological parameters and four site parameters for H_2000 and F_2100 (RCP2.6 and RCP8.5 in MIROC5) and their interactions were analyzed. The upper and lower values for sensitivity analysis were selected based on the quantiles of the distributions in the PTDB of four eco-physiological parameters (CN_leaves, CN_fineroot, Max_sc, and SLA), the quantiles of the posterior distribution from BO for VPD_rsc_start, and the quantiles of the normal distribution (0.69 ± 0.29, mean ± standard deviation) assuming 42% as the coefficient of variance with reference to the value for fine-root turnover in *Picea abies* reported by Brunner et al. (2013). The sensitivity of four site parameters—namely, soil thickness (±25%), N deposition (±25%), shortwave albedo (0.15 ± 0.0375), and the cutting ratio of existing forest (90 ± 9%)—and their interactions with eco-physiological parameters were analyzed.

Reference: Brunner I, Bakker MR, Björk RG, Hirano Y, Lukac M, Aranda X, et al. Fine-root turnover rates of European forests revisited: an analysis of data from sequential coring and ingrowth cores. Plant Soil. 2013;362(1-2): 357–372.

Result

The sensitivity of NPP to eco-physiological parameters was generally higher than that to site parameters (S8 Table). Low CN_leaves (increase in N content of leaves) values, high CN_fineroot (decrease in N content of fine roots) values, and high turnover of fine roots (more rapid turnover) had positive effects on NPP for H_2000, whereas a high SLA (increase in SLA) had a strong negative effect (S8 Table). The positive effects of CN_leaves and turnover of fine roots on NPP for F_2100 decreased under RCP8.5, whereas those related to decreases in Max_sc (reduced Max_sc) and SLA (reduction in SLA) became significant.

Among site parameters, a negative effect on NPP was only found for reduced soil thickness. The effects of other site parameters, such as the cutting ratio of existing forest, N deposition, and shortwave albedo, were quite small.

S8 Table. Sensitivity of the mean value of modeled NPP to site and eco-physiological parameters

Climate	Site parameters		Control_ep	CN_leaves (gC gN^{-1})		CN_fineroot (gC gN^{-1})		Max_sc (m s^{-1})		SLA (m^2 kgC^{-1})		TO_fineroot (year^{-1})		VPD_rsc_start (Pa)	
				33	42	50	99	0.0021	0.0049	7.6	14.0	0.49	0.89	125	206
H_2000	Control_site			+	−−	−	+	−	−		−−−	−	+		
	Cutting ratio	99%		+	−−	−−	+	−	−		−−−	−	+		
		81%		++	−−	−	+	−	−		−−−	−	+		
	N deposition	+25%		+	−−	−	+	−	−		−−−	−	+		
		−25%		+	−−	−	+	−	−−		−−−	−−	+		
	Soil thickness	+25%		++	−−	−	+	−	−		−−−	−	+		
		−25%	−	+	−−	−−		−−	−−		−−−	−−			−
	Shortwave albedo	0.1875		+	−−	−−	+	−−	−		−−−	−−	+		
		0.1125		+	−−	−	+	−	−	+	−−−	−	+		
F_2100	Control_site			+	−	−	+		−−	++	−−−	−			
(RCP2.6)	Cutting ratio	99%			−	−	+		−−	++	−−−	−			
		81%		+	−	−	+		−−	++	−−−	−			
	N deposition	+25%		+	−	−	+		−−	++	−−−	−	+		
		−25%			−	−	+		−−	++	−−−	−			
	Soil thickness	+25%		+	−	−	+		−	++	−−−	−	+		
		−25%			−−	−−			−−	++	−−−	−−			−
	Shortwave albedo	0.1875			−	−	+		−−	++	−−−	−			
		0.1125		+	−	−	+		−−	++	−−−	−	+		
F_2100	Control_site					−		+	−−	+++	−−−				
(RCP8.5)	Cutting ratio	99%				−	+	+	−−	+++	−−−				
		81%				−		+	−−	+++	−−−				
	N deposition	+25%				−	+	+	−−	+++	−−−				
		−25%				−		+	−−	+++	−−−	−			
	Soil thickness	+25%					+	++	−	+++	−−−		+		
		−25%	−		−	−			−−	+++	−−−	−		−	−
	Shortwave albedo	0.1875				−		+	−−	+++	−−−	−			
		0.1125				−	+	+	−−	+++	−−−				

Control_ep, calculation based on control of eco-physiological parameters (STEP-2); Control_site, calculation based on control of site parameters; TO_fineroot, turnover of fine roots. The values for eco-physiological parameters were determined using the 25th and 75th quantiles of their distributions. The symbols ---, --, and - indicate ratios of mean NPP (rNPP) to the combination of control_ep and control_site for rNPP <0.90, 0.90 ≤ rNPP < 0.95, and 0.95 ≤ rNPP < 0.98, respectively, whereas +++, ++, and + represent rNPP ≥ 1.1, 1.05 ≤ rNPP < 1.1, and 1.02 ≤ rNPP < 1.05, respectively. The climate scenario is based on MIROC5.

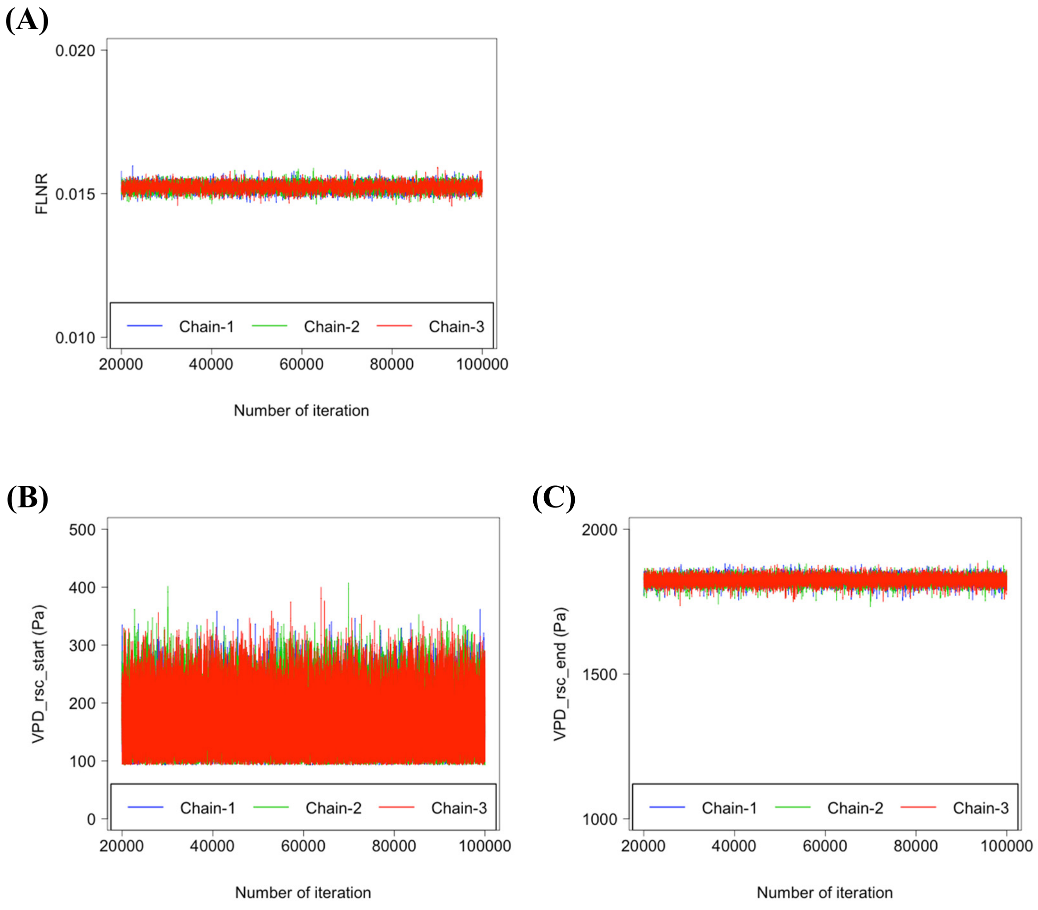

S9 Fig. Markov chain Monte Carlo (MCMC) sampling of three eco-physiological parameters

(A) Fraction of leaf nitrogen in Rubisco (FLNR), and VPD values for (B) starting and (C) ending the reduction of stomatal conductance (VPD_rsc_start and VPD_rsc_end, respectively). Three chains of MCMC sampling are starting from three different initial values.
